# Large-scale Metabolomic Analysis Reveals Potential Biomarkers for Early Stage Coronary Atherosclerosis

**DOI:** 10.1038/s41598-017-12254-1

**Published:** 2017-09-18

**Authors:** Xueqin Gao, Chaofu Ke, Haixia Liu, Wei Liu, Kang Li, Bo Yu, Meng Sun

**Affiliations:** 1 Department of Cardiology, The Second Affiliated Hospital of Harbin Medical University, and The Key Laboratory of Myocardial Ischemia, Chinese Ministry of Education, Harbin, 150081 P. R. China; 2 Department of Epidemiology and Biostatistics, School of Public Health, Medical College of Soochow University, Suzhou, 215123 China; 3 Department of Epidemiology and Biostatistics, School of Public Health, Harbin Medical University, Harbin, 150081 P. R. China

## Abstract

Coronary atherosclerosis (CAS) is the pathogenesis of coronary heart disease, which is a prevalent and chronic life-threatening disease. Initially, this disease is not always detected until a patient presents with seriously vascular occlusion. Therefore, new biomarkers for appropriate and timely diagnosis of early CAS is needed for screening to initiate therapy on time. In this study, we used an untargeted metabolomics approach to identify potential biomarkers that could enable highly sensitive and specific CAS detection. Score plots from partial least-squares discriminant analysis clearly separated early-stage CAS patients from controls. Meanwhile, the levels of 24 metabolites increased greatly and those of 18 metabolites decreased markedly in early CAS patients compared with the controls, which suggested significant metabolic dysfunction in phospholipid, sphingolipid, and fatty acid metabolism in the patients. Furthermore, binary logistic regression showed that nine metabolites could be used as a combinatorial biomarker to distinguish early-stage CAS patients from controls. The panel of nine metabolites was then tested with an independent cohort of samples, which also yielded satisfactory diagnostic accuracy (AUC = 0.890). In conclusion, our findings provide insight into the pathological mechanism of early-stage CAS and also supply a combinatorial biomarker to aid clinical diagnosis of early-stage CAS.

## Introduction

Coronary atherosclerosis (CAS) is a chronic and complicated metabolic disease, and is the pathogenesis of coronary heart disease (CHD). It is characterized by endothelial dysfunction and chronic inflammation that interact with metabolic changes to trigger, propagate and activate lesions in the vessel walls^1,2^. Despite significant progress in the treatment of atherosclerosis (AS), this disease and its complications remain the leading cause of mortality and morbidity worldwide^3,4^. This is mainly because of a lack of effective detection methods for CAS in its early stages, and a poor understanding of the pathophysiology of the disease. Currently, coronary artery angiography (CAG) is the first choice for diagnosis of CAS^5^, but its invasive nature and high cost mean it is not widely used in clinical diagnosis or for tracking progression of the disease. It is generally only used when clinical and biochemical factors clearly indicate the presence of CAS, and its usefulness for prevention is limited^5^.

In the past decade, biomarker discovery has attracted a great deal of interest in CHD research. Biomarkers can be used for early diagnosis and to increase our understanding of disease mechanisms, possibly leading to better clinical decision making in prevention and treatment. Certain biochemical markers for this disease, such as C-reactive protein, have proven useful but have limitations^6^.

Metabolomics, the global quantitative measurement of low molecular weight endogenous metabolites in tissues or biological fluids (serum, plasma, urine), is promising for discovery of novel biomarkers and increasing understanding of the complex process of CAS development^7–10^. The number of studies using non-targeted metabolomics for study of cardiovascular disease is ever-increasing, and has included studies of myocardial ischemia^11,12^, acute coronary syndrome^13,14^, heart failure^15^, and hypertension^16^. Recently, metabolomics has been successfully used to identify new biomarkers and characterize metabolic changes associated with AS^17–21^. Sun *et al*.^18^ and Mariona *et al*.^19^ used liquid chromatography/mass spectrometry (LC-MS) to study AS-based animal models, and revealed metabolic abnormalities for lipids, bile acids, and fatty acids. However, there is some controversy regarding the validity of extrapolation of animal model results to humans. Chen *et al*.^20^ successfully discriminated AS patients from healthy controls using metabolomic profiling and found that perturbations of fatty acids involved in the development of AS, especially palmitate, could be used as plasma biomarkers for atherosclerosis. Using gas chromatography/mass spectrometry and nuclear magnetic resonance spectroscopy for metabolomics, Joanna *et al*.^21^ found that at least 24 metabolites were significantly modified in a group of AS patients, and most of the changes were related to insulin resistance. However, they used healthy people as the controls and both of the groups were limited in size, which could result in false and nonspecific results. In addition, to date, few studies have focused on metabolic markers to identify early stage CAS patients.

In this study, 60 early stage CAS patients and 60 CAG-defined controls were used to detect metabolic signatures of early stage CAS based on liquid chromatography quadrupole time-of-flight mass spectrometry (LC-QTOF/MS) metabolomics. Subsequently, we identified potential biomarkers and explored their related metabolic pathways. The metabolites and their pathways might serve as targets for therapeutic intervention or prevention. Finally, a second study was performed in an independent population (40 patients with early stage CAS and 40 controls) to validate the discrimination performance of the selected metabolites, and confirm their potential as diagnostic indicators.

## Results

### Characteristics of patients

A total of 200 patients (100 early CAS patients and 100 controls) confirmed by CAG served as the study population. Based on the time they entered the current study, the enrolled subjects were separated into a training set (60 patients and 60 controls) for biomarker selection and establishment of a model for discrimination of early CAS patients and controls, and a test set (the remaining cases) for validation of the model. Clinical characteristics and CAG parameters for these participants are summarized in Table 1. By design, all early stage CAS patients had mild angiographically documented coronary stenosis in at least one major coronary artery, with a mean percentage of 36.53 ± 12.82% in the training set, and 42.15 ± 11.76% in the test set. By contrast, no controls showed apparent lesions in CAG.

**Table 1:** Demographic and clinical characteristics of early stage CAS patients and their controls in the training and test set.

	Training set			Test set		
	early CAS (n = 60)	Control (n = 60)	*P*-value	early CAS (n = 40)	Control (n = 40)	*P*-value
Male	37(61.67)	29(48.33)	0.1421	17(42.50)	20(50.00)	0.5011
Age (years)	58.43 ± 9.69	55.88 ± 9.50	0.1480	61.65 ± 9.78	57.98 ± 7.16	0.0588
Weight (kg)	70.03 ± 10.41	69.21 ± 10.70	0.6910	66.77 ± 11.70	68.45 ± 10.02	0.5155
Smoking Current	17(28.33)	14(23.33)	0.0623	15(37.50)	13(32.50)	0.1852
Former	13(21.67)	5(8.33)		5(12.50)	1(2.50)	
Never	30(50.00)	41(68.33)		20(50.00)	26(65.00)	
Hypertension	27(45.00)	19(31.67)	0.1331	23(57.50)	13(32.50)	0.0246
Hyperlipidemia	7(11.67)	4(6.67)	0.3426	7(17.50)	4(10.00)	0.3301
Prior coronary artery disease	2(3.33)	0(0.00)	0.5587	4(10.00)	2(5.00)	0.3959
TC (mmol/L)	4.95 ± 1.42	4.69 ± 0.91	0.2450	4.97 ± 1.22	4.76 ± 1.10	0.4410
TG (mmol/L)	2.20 ± 3.01	2.10 ± 1.07	0.8162	2.06 ± 1.04	1.99 ± 1.45	0.8086
LDL (mmol/L)	2.53 ± 0.77	2.51 ± 0.76	0.9205	2.73 ± 0.93	2.58 ± 0.57	0.3752
HDL (mmol/L)	1.25 ± 0.40	1.38 ± 0.37	0.0748	1.37 ± 0.55	1.48 ± 0.87	0.4924
apoA (g/L)	1.00 ± 0.21	1.14 ± 0.22	0.0018	1.04 ± 0.16	1.19 ± 0.22	0.0014
apoB (g/L)	0.77 ± 0.24	0.69 ± 0.18	0.0740	0.87 ± 0.29	0.73 ± 0.18	0.0174
hs-CRP (mg/L)	2.72 ± 3.03	2.05 ± 2.14	0.2635	5.18 ± 9.16	1.79 ± 1.99	0.0287
CK (IU/L)	81.24 ± 50.78	79.22 ± 40.10	0.8112	71.41 ± 32.63	64.38 ± 33.64	0.3522
CK-MB (IU/L)	1.47 ± 3.67	0.99 ± 2.20	0.3951	0.50 ± 0.53	0.47 ± 0.42	0.8332
Troponin I (μg/L)	0.39 ± 2.23	0.19 ± 1.03	0.5523	0.03 ± 0.05	0.02 ± 0.04	0.7098
Ejection fraction (%)	65.49 ± 7.24	65.51 ± 6.60	0.9854	63.26 ± 6.54	63.41 ± 7.23	0.9274
Coronary angiography parameters						
Number of artery stenosis	1.95 ± 0.81	—	—	2.38 ± 0.77	—	—
Percentage of stenosis (%)	36.53 ± 12.82	—	—	42.15 ± 11.76	—	—

Values are presented as mean ± SD or number (%); TC, Total Cholesterol; TG, Triglyceride; LDL, Low Density Lipoprotein; HDL, High Density Lipoprotein; apoA, Apolipoprotein A; apoB, Apolipoprotein B; hs-CRP, hypersensitive C-reactive protein; CK, Creatine Kinase.

### Metabolic profiling of the plasma samples

We obtained 3892 and 2936 aligned individual peaks (variables) in ESI+ and ESI−mode, respectively. These peaks were for quasi-molecular ions, isotope ions, adduct ions, and fragment ions of the metabolites. Examples of the LC-QTOF/MS total ion chromatograms of plasma samples from an early stage CAS patient and a control subject are shown in Supplementary Fig. S1.

Firstly, the unbiased PCA revealed that all the QC samples were tightly clustered in PCA score plots (Supplementary Fig. S2), which confirmed that our method was robust. The score plots from PCA model performed on all plasma samples also showed that there were no extreme outliers that needed to be excluded from subsequent analysis. Nonetheless, no obvious separation trends between the two groups were observed when variables were not selected.

**Figure 1:**
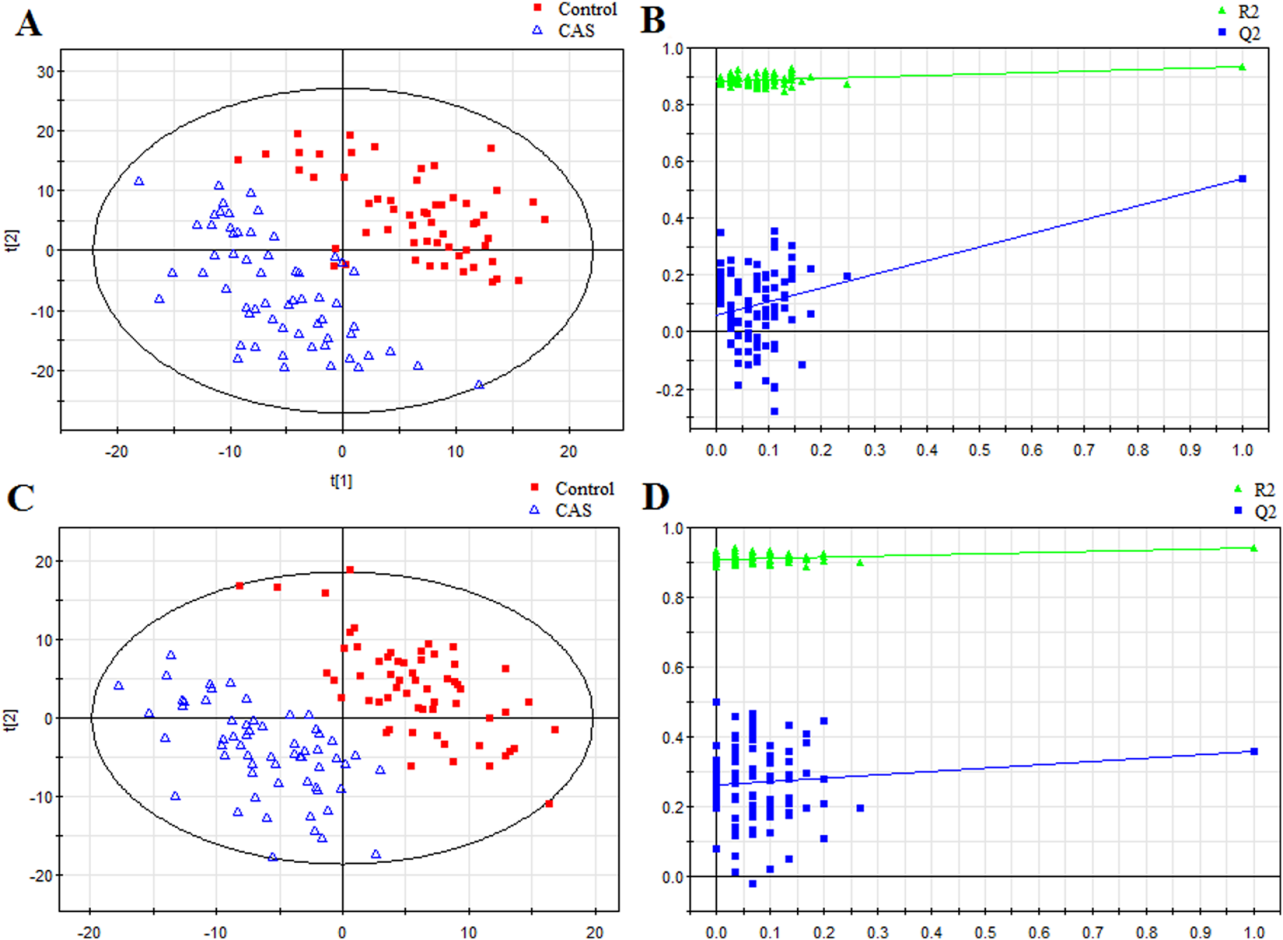
PLS-DA score plots and validation plots for discriminating early-stage CAS patients from controls. (**A**) PLS-DA score plot in ESI+ mode; (**B**) validation plot in ESI+ mode; (**C**) PLS-DA score plot in ESI− mode; (**D**) validation plot in ESI− mode.

Then, to further explore the metabolic differences between the early stage CAS group and the controls, PLS-DA models were established in the training set. As shown in Fig. 1A,C, the early stage CAS subjects were obviously separated from the controls with little overlap. The values of those parameters quantifying the PLS-DA model were positive (R^2^*X* = 0.178, R^2^*Y* = 0.933, Q^2^ = 0.540 in ESI+ mode and R^2^*X* = 0.168, R^2^*Y* = 0.943, Q^2^ = 0.356 in ESI−mode), indicating the goodness of fit and prediction ability of the model^22^. In the training set, three samples from control subjects (5%) were wrongly classified in ESI+ mode, while no misclassifications were found in ESI−mode. Furthermore, the supervised PLS-DA models were validated with permutation tests to ensure those models were not overfitted. The validation plots of permutation tests (Fig. 1B,D) supported the validity of these constructed PLS-DA models, as all the values of the goodness of fit (R^2^ and Q^2^) calculated from the permuted data (in green on the left) were lower than the original point on the right, and the Q^2^ regression line (in blue) had a negative intercept^23^.

### Selection and identification of potential metabolic biomarkers

To identify potential biomarkers of early stage CAS, variables that dominated the discrimination were first selected according to their VIP values (VIP > 1), which were calculated from the PLS-DA model. A nonparametric Kruskal-Wallis test was then performed, and variables without significant differences between the two groups (*p* ≥ 0.05) were eliminated. The remaining biomarker candidates were selected for subsequent identification. The procedure for metabolite identification is detailed in our previous work^23^. Following the procedure, a total of 20 differential endogenous metabolites in ESI+ mode and 22 metabolites in ESI−mode were identified (Table 2). Among them, the identities of seven were confirmed using reference standards, and 29 were identified by online database searches (HMDB and METLIN) and LC-QTOF/MS. The MS/MS spectrums of the metabolites are shown in the Supplementary Figs S3–S7.

**Table 2:** Plasma metabolic biomarkers for discriminating early-stage CAS patients from controls.

Marker	Identity	RT(min)	*m/z*	Adduction	ppm	*VIP* ^a^	*p*-Value^b^	FC^c^	AUC	RSD (%)^d^	Pathway
Positive electrospray ionization mode (ESI+)											
V01	LysoPC(18:1)^f^	15.92	544.3365	[M + Na]^+^	1	1.7714	0.0272	−0.11	0.6184	9.75	Phospholipid metabolism
V02	LysoPC(18:4(6Z,9Z,12Z,15Z))^f^	13.73	516.3039	[M + H]^+^	8	1.8637	0.0114	−0.18	0.6357	8.39	Phospholipid metabolism
V03	LysoPC(20:4)^f^	14.31	544.3350	[M + H]^+^	8	1.7796	0.0031	−1.17	0.6588	23.72	Phospholipid metabolism
V04	LysoPC(16:0)^f^	15.53	496.3382	[M + H]^+^	3	1.9185	0.0169	−0.1	0.6281	12.83	Phospholipid metabolism
V05	LysoPC(22:4(7Z,10Z,13Z,16Z))^f^	16.03	572.3681	[M + H]^+^	5	1.6443	0.0131	−0.22	0.6330	11.38	Phospholipid metabolism
V06	LysoPC(15:0)^f^	16.53	504.3072	[M + Na]^+^	2	1.5424	0.0061	−0.17	0.6471	5.32	Phospholipid metabolism
V07	PE(16:0/0:0)^f^	14.40	454.2939	[M + H]^+^	2	1.7497	0.0030	−0.89	0.6594	29.05	Phospholipid metabolism
V08	LysoPE(18:2)^f^	13.74	478.2930	[M + H]^+^	0	1.7939	0.0141	−0.18	0.6316	7.06	Phospholipid metabolism
V09	PG(18:0/0:0)^f^	13.69	513.3240	[M + H]^+^	10	1.2430	0.0021	−0.37	0.6649	23.05	Phospholipid metabolism
V10	PC(14:0/14:0)^f^	18.21	678.4994	[M + H]^+^	10	1.3851	0.0013	0.32	0.6728	20.96	Phospholipid metabolism
V11	PE(P−16:0/0:0)^f^	15.14	438.2969	[M + H]^+^	2	1.2223	0.0261	−0.15	0.6193	18.77	Phospholipid metabolism
V12	PE(42:8)^f^	19.20	833.5826	[M + NH4]^+^	2	1.8696	0.0348	−0.21	0.6132	17.69	Phospholipid metabolism
V13	Phytosphingosine^f^	14.01	318.2992	[M + H]^+^	3	1.7031	0.0288	0.48	0.6173	23.43	Sphingolipid metabolism
V14	Sphinganine^f^	13.65	302.3050	[M + H]^+^	1	1.6431	0.0064	0.31	0.6462	4.63	Sphingolipid metabolism
V15	MG(18:2)^f^	18.18	372.3085	[M + NH4]^+^	6	1.9830	0.0001	1.19	0.7070	23.76	Phospholipid metabolism
V16	MG(18:3)^f^	19.20	353.2648	[M + H]^+^	10	1.3125	0.0049	0.55	0.6509	20.47	Phospholipid metabolism
V17	MG(18:1)^f^	19.48	357.3016	[M + H]^+^	4	1.5380	0.0008	0.66	0.6804	10.59	Phospholipid metabolism
V18	DG(36:3)^f^	21.50	657.4790	[M + K]^+^	9	1.6150	0.0317	−0.38	0.6152	20.11	Phospholipid metabolism
V19	DG(38:5)^f^	23.32	643.5240	[M + H]^+^	8	1.5130	0.0448	−0.53	0.6076	29.40	Phospholipid metabolism
V20	Docosahexaenoic acid^*e*^	18.83	329.2445	[M + H]^+^	9	1.2292	0.0363	0.46	0.6123	27.33	Fatty acids metabolism
Negative electrospray ionization mode (ESI−)											
V21	LysoPC(18:1)	16.03	556.3156	[M + Cl]^−^	3	1.5944	0.0230	−0.11	0.6165	19.68	Phospholipid metabolism
V22	LysoPC(18:2(9Z,12Z))	14.47	554.2992	[M + Cl]^−^	4	1.8542	0.0284	−0.11	0.6123	17.03	Phospholipid metabolism
V23	LysoPC(22:6(4Z,7Z,10Z,13Z,16Z,19Z))	14.36	566.3261	[M − H]^−^	1	1.9864	0.0167	−0.12	0.6226	5.24	Phospholipid metabolism
V24	7Z,10Z,13Z,16Z,19Z-docosapentaenoic acid^f^	19.19	329.2455	[M − H]^−^	9	2.2605	0.0105	0.49	0.6311	8.48	Fatty acids metabolism
V25	Eicosatrienoic acid^f^	19.64	305.2464	[M − H]^−^	7	1.9257	0.0270	0.25	0.6133	17.04	Fatty acids metabolism
V26	Linoelaidic Acid^*e*^	19.23	279.2300	[M − H]^−^	10	2.5287	0.0046	0.37	0.6450	10.35	Fatty acids metabolism
V27	Linolenic Acid^*e*^	18.30	277.2165	[M − H]^−^	2	2.3205	0.0022	0.48	0.6567	6.52	Fatty acids metabolism
V28	Pentadecanoic acid^f^	19.39	241.2158	[M − H]^−^	6	1.8329	0.0193	0.26	0.6199	22.49	Fatty acids metabolism
V29	Elaidic Acid^*e*^	20.39	281.2469	[M − H]^−^	6	2.3784	0.0145	0.39	0.6252	14.41	Fatty acids metabolism
V30	Myristic acid^e^	18.49	227.2006	[M − H]^−^	4	1.7568	0.0433	0.35	0.6035	14.21	Fatty acids metabolism
V31	Δ2-trans-Hexadecenoic Acid^f^	18.83	253.2164	[M − H]^−^	3	2.0748	0.0270	0.47	0.6133	6.42	Fatty acids metabolism
V32	Palmitic acid^e^	20.22	255.2316	[M − H]^−^	5	2.3728	0.0112	0.39	0.6299	20.57	Fatty acids metabolism
V33	Eicosadienoic acid^*f*^	20.65	307.2624	[M − H]^−^	6	2.2876	0.0132	0.3	0.6269	18.87	Fatty acids metabolism
V34	3-Hydroxycapric acid^*f*^	11.02	187.1332	[M − H]^−^	4	1.6750	0.0480	0.25	0.6013	13.08	Fatty acids metabolism
V35	Prasterone sulfate^*f*^	10.23	367.1571	[M − H]^−^	3	1.8665	0.0112	−0.56	0.6299	10.49	Steroid hormone biosynthesis
V36	L-Fucose^*e*^	0.92	199.0383	[M + Cl]^−^	2	1.7762	0.0084	−0.39	0.6350	29.22	Fructose and mannose metabolism
V37	Dihydro-3-coumaric acid^f^	16.11	165.0540	[M − H]^−^	10	1.6107	0.0176	0.69	0.6216	23.79	Phenylalanine metabolism
V38	p-Tolyl Sulfate^*f*^	6.45	187.0065	[M − H]^−^	2	1.4817	0.0216	0.8	0.6177	15.9	Gut microbial metabolism
V39	Indoxylsulfuric acid^*f*^	5.52	212.0045	[M − H]^−^	10	1.9667	0.0263	0.35	0.6138	13.56	Tryptophan metabolism
V40	3-oxo-tetradecanoic acid	14.45	241.1799	[M − H]^−^	4	1.7352	0.0079	0.65	0.6360	16.35	Fatty acids metabolism
V41	Hexadecadienoic acid	17.52	251.1990	[M − H]^−^	10	1.4676	0.0035	0.45	0.6497	14.91	Fatty acids metabolism
V42	3-oxo-dodecanoic acid	12.18	213.1489	[M − H]^−^	3	1.7331	0.0369	0.3	0.6069	11.6	Fatty acids metabolism

Abbreviations: Retention time (RT, min); Measured mass to charge ratio (*m/z*); Variable importance in the projection (VIP); Fold change (FC); Mass error (ppm); The area under the ROC curve (AUC); Relative standard deviation (RSD%).

^a^Variable importance in the projection (VIP) was obtained from PLS-DA with a threshold of 1.0.

^b^The *p*-value was calculated from the nonparametric Kruskal-Wallis rank sum test.

^c^Fold change was calculated as a binary logarithm of the arithmetic mean ratio between patients vs controls, where a positive value indicates that a relatively higher concentration present in patients while a negative value means a relatively lower concentration as compared to the control subjects.

^d^Variation of the biomarker concentrations in the quality control samples expressed as relative standard deviation (RSD%).

^e^The metabolite was verified by reference standard.

^f^The metabolite was identified by online database.

The concentrations of 24 metabolites were significantly higher in the early stage CAS patients than the controls (Table 2). By contrast, the concentrations of 18 metabolites were lower in early stage CAS patients than the controls. These differences between the two groups are expressed as fold change. In addition, the relative standard deviations of the intensities of these 42 biomarkers were calculated in the QC samples, and varied from 4.63% to 29.40% with a median of 16.14%. These data indicated that our metabolic profiling platform was robust, and that changes in the biomarkers arose from the disease state rather than analytical errors.

Furthermore, to investigate whether these differential metabolites are closely associated with clinical measures, Pearson correlation analysis was performed. However, the early stage CAS patients and their controls enrolled in this study exhibited no significant differences in clinical characteristics, so it is to be expected that no correlation was found between the majority of metabolic biomarkers and clinical parameters. Although certain biomarkers showed correlations with clinical parameters at a cutoff point of *p* = 0.05 (e.g. LysoPC(18:4(6Z,9Z,12Z,15Z)) and LysoPE(18:2)), the correlation coefficients were very low (<0.3075), as presented in Supplementary Table S1. Thus, the established correlations needed to be further investigated.

### Evaluation of the Diagnosis Potential of the Metabolic Biomarkers

To assess the diagnostic utility of the metabolites for discrimination between early stage CAS patients and controls, ROC curves were constructed for the 42 metabolites. For most biomarkers, the value of the area under the curve (AUC) was < 0.7 (Table 2), indicating they had poor prediction ability. Therefore, multiple metabolites will need to be combined to diagnose early CAS.

**Figure 2:**
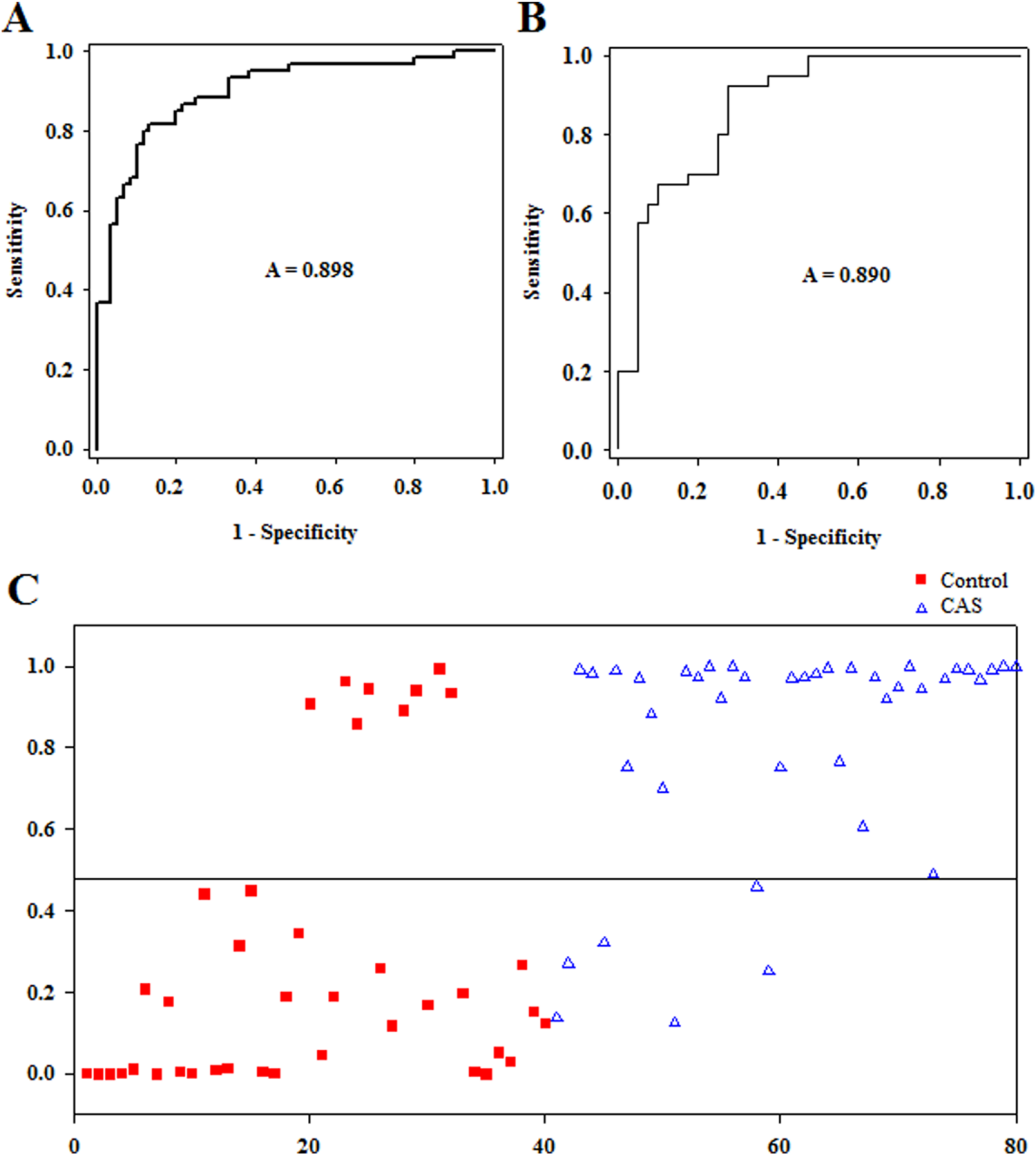
ROC curves of the combined plasma biomarkers for distinguishing early-stage CAS patients from controls in (**A**) the training set; (**B**) the test set. (**C**) Predictive scores plot of plasma samples between patients and their controls in the test set.

**Figure 3:**
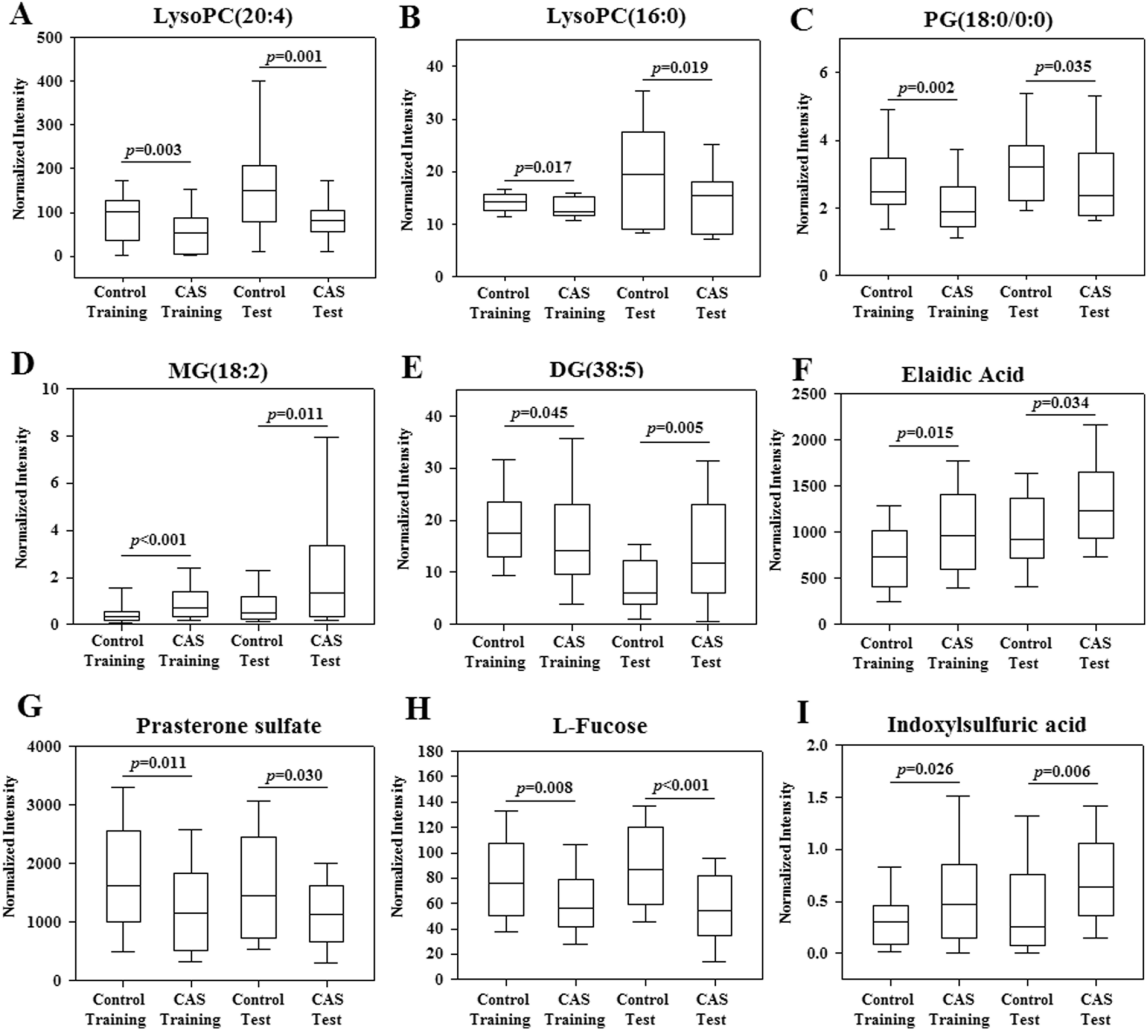
Bar plots of potential biomarkers. Value in the box plots are shown as the normalized peak areas of the metabolites. The horizontal line inside the box is the median, and the bottom and top boundaries of the boxes are the 25th and 75th percentiles, respectively. Lower and upper whiskers are the 5th and 95th percentiles, respectively.

A binary logistic regression model was constructed based on the 42 identified biomarkers in the training set. Through a forward stepwise variable selection analysis (Wald test), nine metabolites (i.e. lysophosphatidylcholine (LysoPC) (20:4), LysoPC(16:0), phosphatidylglycerol (18:0/0:0), elaidic acid, prasterone sulfate, l-fucose, monoglyceride (MG) (0:0/18:2(9Z,12Z)/0:0), diglyceride (DG) (20:2(11Z,14Z)/18:3(9Z,12Z,15Z)/0:0), and indoxylsulfuric acid) were selected as the best predictors for early CAS discrimination. The prediction model was established as follows: probability = 1/[1 + exp(−(6.297 − 0.014 × V03 − 0.265 × V04 − 0.322 × V09 + 0.636 × V15 − 0.076 × V19 + 0.002 × V29 − 0.001 × V35 − 0.024 × V36 + 2.929 × V39))]. As expected, the combinatorial model yielded a satisfactory result, with an AUC of 0.898 (95% confidence interval 0.841–0.955) (Fig. 2A). Using the best cutoff value of 0.4789, the sensitivity and specificity were 86.7% and 81.7%, respectively. The relative concentrations of these nine plasma metabolite biomarkers across all groups are presented in Fig. 3. Compared with the control group, three metabolites (MG(0:0/18:2(9Z,12Z)/0:0), elaidic acid, and indoxylsulfuric acid) showed increased concentrations in the patient group, whereas the other six metabolites (e.g., LysoPC(20:4) and l-fucose) showed decreased levels.

To validate the diagnostic capability of the combinatorial model, an independent cohort of 40 early stage CAS patients and 40 control subjects was used. None of the samples had been previously included in the training set, and this allowed for estimation of true predictive accuracy. In this case, the plasma biomarkers model still exhibited good classification ability (Fig. 2B). The AUC reached 0.890 (95% confidence interval 0.822–0.961). The percentage of correct diagnoses at the same cutoff value of 0.4789 was 85.0% for early stage CAS patients and 80.0% for the control subjects (Fig. 2C). The external validation study confirms the outstanding performance of the LC-MS plasma metabolomics platform for diagnosis of early stage CAS patients.

### Staging analysis

In addition to accurate diagnosis, classification of the severity of CAS is critical for patient management and determining prognosis. Thus, we attempted to investigate the potential of plasma metabolomics for stratification of the severity of CAS in combined training and test data sets. For simplicity in this study, we divided the patients into three groups based on the number of artery stenoses. When using the entire data set, the established PLS-DA models exhibited good ability to discriminate from each other (See Supplementary Fig. S8). Furthermore, all AUC values were greater than 0.90 (Table 3). These results highlight the potential of metabolomics in the staging of CAS. In addition, the concentrations of five metabolites were found to be significantly different in patients who were at different stages of the disease (Supplementary Fig. S9). However, further validation and selection of more differential metabolites should be carried out using a larger patient cohort to confirm these results.

**Table 3:** The diagnostic potential of PLS-DA Models for Different CAS stages.

Positive mode	Group1/2	Group1/3	Group2/3
AUC	0.971	0.987	1.000
Sensitivity(%)	100.0	94.4	100.0
specificity(%)	83.3	96.7	100.0
**Negative mode**	**Group1/2**	**Group1/3**	**Group2/3**
AUC	0.945	0.909	0.923
sensitivity(%)	90.9	94.4	91.7
specificity(%)	80.6	71.0	87.9

## Discussion

Complex diseases such as CAS with multiple etiological factors necessitate a systemic approach for mechanistic understanding and optimization of early diagnosis. In this study, we applied metabolomics, covering thousands of small molecular endogenous metabolites, to characterize metabolic alterations of early stage CAS.

Our results demonstrated that multivariate models can accurately distinguish early stage CAS patients from control subjects, and 42 plasma metabolites were identified as biomarkers of early stage CAS. However, diagnosis based on so many metabolites would not be convenient or economical in clinical practice. Thus, simplification of the plasma metabolite signature is required for practical diagnosis of early stage CAS. To accomplish this, we performed a binary logistic regression, in which nine metabolites were selected as the best predictors for early stage CAS discrimination. Furthermore, the AUC was calculated to quantitatively assess the diagnostic performance of this simplified metabolite signature. The findings indicated that the simplified metabolite signature of the nine biomarkers was a good classifier for discrimination of early stage CAS patients from controls, and this was supported by the satisfactory AUC values of 0.898 in the training set and 0.890 in the test samples. However, further studies involving a larger sample set or heterogeneous population are needed to verify these novel biomarkers.

In the current study, in addition to assessing the potential of these biomarkers as diagnostic indicators for early stage CAS, we investigated the biology and metabolic functions of the biomarkers to enhance our understanding of the disease’s metabolic mechanisms. The pathways for the biomarkers (Table 2) were determined by searching the KEGG PATHWAY Database, Human Metabolome Database and ChEBI Database.

Among the metabolites, a series of LysoPCs and lysophosphatidylethanolamines (LysoPEs), in addition to phosphatidlycholines (PC), phosphatidylethanolamines (PE) and phosphatidylglycerols (PG), were greatly altered in early stage CAS patients compared to the controls. A well-known mechanism of lysophospholipid production is hydrolysis of phosphoglycerides by phospholipase A2 (Supplementary Fig. S10A). Although elevated levels of lysophospholipids have been reported to induce oxidative stress on endothelial cells, which leads to AS and cardiovascular disease^24–26^, it has also been observed that lysophospholipids produced by a PLA2-like activity of Paraoxanase 1 contribute to inhibition of macrophage biosynthesis and consequently reduce cellular cholesterol accumulation and atherogenesis^27^. Lysophospholipids have been widely recognized as pro-inflammatory and pro-atherogenic metabolites^28^, but some recent population-based studies have suggested lysophospholipids have protective effects on CHD and its risk factors. Fernandez *et al*. and Stegemann *et al*. found an inverse association between several LysoPCs and incident CHD^29,30^. In a study of type 2 diabetes, LysoPC 18:2 was found to be inversely associated with incident diabetes and impaired glucose tolerance^31^. Our study confirms and extends on these previous findings. With this knowledge, we inferred that a disturbed phospholipid catabolism would be closely interrelated with early stage CAS.

We found plasma concentrations of three MGs and two DGs, MG(18:2), MG(18:3), MG(18:1), DG(36:3) and DG(38:5), were significantly disrupted in the early stage CAS patients. MG(18:1), MG(18:2) and MG(18:3) were upregulated in the patients compared with controls, while DG(36:3) and DG(38:5) were downregulated relative to the controls. The circulating DGs are mostly derived from phosphatidic acid, and then converted into triglycerides under the catalysis of DG acyltransferase. Triglycerides are further hydrolyzed to release fatty acids and MGs by the action of lipoprotein lipase or hormone sensitive lipase (Supplementary Fig. S10A). Within the intestinal wall, MGs are precursors to triglycerides via the MG pathway before being transported in lymph to the liver^32^. Thus, it has been shown that DGs and MGs are central in the synthesis and breakdown of triglycerides, and a large randomized analysis recently been proved this has a positive causal effect on CHD risk^33^. On the other hand, the disturbed DGs and MGs metabolism observed in this study may lead to an increase in the number of free fatty acids, and further migration and invasion of macrophages via the p38 MAP-kinase signaling pathway, Toll-like receptors 2 and 4, and JNK-dependent pathways^34,35^. Several studies have suggested accumulated macrophages exert a key role in the formation and development of AS^36^. Therefore, the perturbed DG and MG metabolites, particularly MG(18:2) and MG(18:3), should be associated with the onset of early stage AS, which is in line with a previously report that demonstrated they are involved in the pathogenesis of CHD^23,32^.

The levels of phytosphingosine and sphinganine in the early stage CAS patients compared to the controls were elevated significantly in our study. It is known that accumulation of phytosphingosine and sphinganine occurs because of the action of sphingomyelinases hydrolysis of sphingolipids^37^. Sphingolipids, which are a large class of lipids, play important roles as both membrane components and signaling molecules involved in diverse cell processes, including cell-cell interactions, cell proliferation, cell differentiation, and apoptosis^38^. Emerging evidence has shown that sphingolipid-mediated cellular signaling pathways play a critical role in cardiovascular pathophysiology^39^. It has been reported that sphingolipids have the capacity to reduce triglyceride and cholesterol levels^40^. However, higher concentrations of phytosphingosine and sphinganine in plasma samples from patients suggested that sphingolipids were depleted, which increased the risk of AS and metabolic syndrome^41^. In addition, using metabolomics, Liu *et al*. and Qi *et al*.^42,43^ found that phytosphingosine and sphinganine levels were significantly increased in a myocardial ischemia rat model, which is in accordance with our study. Therefore, it is reasonable to suggest that the sphingolipid metabolism is activated in the early stages of CAS.

We found that several metabolites of interest were involved in metabolic processes related to long-chain fatty acids. Generally, fatty acids are an important source of energy for the heart. Under AS conditions, the oxygen requirements of the heart exceed the oxygen supply to the heart. In our study, we observed significantly enhanced levels of plasma long-chain fatty acids in early stage CAS patients, indicating that the increased abundance of plasma long-chain fatty acids was probably the result of strong de novo fatty acid synthesis during the initiation and progression of AS to supply the required energy. Zha *et al*.^44^ showed that syntheses of polyunsaturated fatty acids and unsaturated fatty acids were significantly upregulated in an early AS animal model. Interestingly, we also found that the plasma concentrations of long-chain fatty acids, such as palmitic acid, linolenic acid, and elaidic acid, were significantly higher in the early stage CAS group than the control group. Therefore, the metabolism of long-chain fatty acids might have a pivotal pathogenetic role in triggering CAS.

In summary, our study demonstrates that LC-MS-based plasma metanolomics is a powerful approach that can accurately distinguish early stage CAS patients from control subjects. The results provide a panel of metabolite markers that have clinical potential for disease diagnosis and patient stratification for early CAS. These metabolite markers are involved in several key metabolic pathways such as phospholipid metabolism, sphingolipid metabolism, and fatty acid metabolism. The present study is the first clinic metabolomics study focusing on early stage CAS to suggest that plasma metabolomics could be used for non-invasive early diagnosis and surveillance of CAS with high sensitivity and specificity. The elucidation of the associations between biomarkers and early stage CAS events increases mechanistic understanding of early stage CAS.

## Methods

### Patients

The study protocol was approved by the Ethics Committee of the Second Affiliated Hospital of Harbin Medical University. All experiments were performed in accordance with relevant guidelines and regulations. Subjects were enrolled between August, 2012 and July, 2014 from the Department of Cardiology, 2nd Affiliated Hospital of Harbin Medical University, Harbin, China. Patients were included in this study if they underwent diagnostic CAG for the evaluation of coronary artery disease and did not have significant coronary artery stenoses (i.e., stenosis < 50%). According to Tousoulis’s study^45^, we defined early stage CAS patients as individuals with newly diagnosed, angiographically documented coronary stenosis < 50% in at least one major coronary artery, while the controls showed no apparent lesions in angiography. Exclusion criteria for this study included the following: previous myocardial infarction or myocardial revascularization or percutaneous coronary intervention; heart failure (left ventricular ejection fraction less than 30%); valvular heart disease; any metabolic disease (e.g., diabetes mellitus); malignancy; liver/renal disease; inflammatory disease (e.g., infections); pregnancy or lactation; multiple organ function failure; and previous coronary artery bypass surgery. All participants provided written informed consent, were screened for age, sex, weight, cardiac risk factors, prior cardiac disease, cardiac medications, and were given hematological and biochemical examinations.

Peripheral venous blood samples (5 mL) were collected in the morning before breakfast from 100 early stage CAS patients and 100 controls using vacutainer tubes containing fresh sodium dihydrogen phosphate anticoagulant. The plasma samples were separated by centrifugation at 1000 × *g* for 10 min and stored at -80 °C until required for further analysis.

### Blank and quality control samples

Blank and quality control (QC) samples were analyzed throughout the whole experimental procedure. A blank (75% acetonitrile) was run after every five samples to identify and minimize sample carryover. The QC samples were created by combining equal volumes of plasma samples from 20 patients with early stage CAS and 20 controls. The QC samples were injected four times in randomized order within every analytical batch, and used to monitor the stability and performance of the system and evaluate the quality of the acquired data.

### Sample preparation

Before analysis, all of the plasma samples, including the QC samples, were processed according to our previous method with minor modifications^23^. Briefly, methanol (1000 μL) was added to 200 μL of plasma and vortex-mixed vigorously for 2 min. The mixture was centrifuged at 14,000 × *g* for 15 min at 4 °C. The supernatant was transferred to a clear vial and reduced under a stream of nitrogen at 37 °C. The residue was dissolved in 200 μL of acetonitrile/water (3:1/v:v), vortex-mixed for 60 s and then centrifuged at 14,000 × *g* for 15 min at 4 °C. The supernatant was then placed into a sample vial for LC-QTOF/MS analysis.

### Chromatography

Chromatography separation was performed on an Agilent Technologies 1260 liquid chromatography system using a ZORBAX SB-C18 column (100 mm × 3.0 mm i.d., 1.8 μm, Agilent, Santa Clara, CA) at 40 °C. The mobile phase was a mixture of water containing 0.1% formic acid (A) and acetonitrile with 0.1% formic acid (B). The mobile phase flow rate was 0.5 mL/min. A linear gradient elution was performed, starting with 5% B, increasing to 98% B over 18 min, and was holding at 98% B for 3 min. Subsequently, the mobile phase was returned to the initial condition (5% B) within 0.1 min, and maintained at this level for 7 min for equilibration. The injection volume of the sample was 10 μL. All samples were maintained at 4 °C during the analysis^46^.

### Mass spectrometry

Metabolic profiling was conducted using an Agilent 6530 series quadrupole time-of-flight mass spectrometer equipped with a dual electrospray ionization source (ESI). The ionization was operated in positive (ESI+) or negative (ESI−) mode. The mass spectrometry parameters were set as previously described^23^. To ensure mass accuracy and reproducibility, the mass spectrometer was internally mass calibrated in real time with purine (*m/z* 121.0509 and *m/z* 119.0363 in ESI + and ESI− mode, respectively).

Tandem mass spectrometry (MS/MS) experiments were carried out in targeted MS/MS mode to identify potential biomarkers. Argon was employed as the collision gas, and collision energy was set at 10, 20, or 40 eV.

### Data preprocessing and annotation

The raw data acquired from LC-QTOF/MS were initially converted into mzData format via Mass Hunter Qualitative Analysis Software (Agilent) and then imported to xcms package in the R platform for preprocessing^47^. The default xcms parameters were used, with the following exceptions: xcmsSet (method = “centWave”, peakwidth = c(10,50))^47^. The preprocessing result was obtained with a three-dimensional data set of the retention time, mass-to-charge ratio (*m/z*), and peak intensity. Then the R package CAMERA was used for annotation of isotope peaks, adducts, and fragments^47^. Finally, the data for each sample were normalized to the total sum of peak intensities before statistical analysis^48^.

### Statistical analysis

Multivariate data analysis was performed using SIMCA-P 11.5 software (Umetrics AB, Umea, Sweden). Unsupervised principal component analysis (PCA) was first carried out with all samples to provide an overview of the grouping trends and outliers^49^. Then, supervised partial least-squares discriminant analysis (PLS-DA) was used to find differences between the early stage CAS patients and controls. Variable importance in the project (VIP) was calculated as a coefficient for selection of variables^50^. To validate the robustness of the supervised model and evaluate the degree of overfitting, permutation tests with 100 iterations were performed^51^. In addition to the multivariate statistical method, the nonparametric Kruskal–Wallis test was also applied to measure the significance of each variable. Only mass features with multivariate and univariate statistical significance (VIP > 1.0 and *p < *0.05) were included in the list of candidate markers contributing most to the discrimination, which was then submitted to the metabolite identification procedure. Receiver operating characteristic (ROC) curve analysis and binary logistic regression were performed using SPSS software (IBM SPSS Statistics 22, USA) following the previously published data analysis method^52^. The training set was used to generate the classification model, and an independent test set was then subjected to the constructed model to evaluate its diagnostic ability.

### Metabolite identification

Markers were identified through a multiple-step procedure. The first step was to find quasi-molecular ions via analysis of the peak list and annotation results and determine the corresponding molecular weights. The second step involved performing the MS/MS experiments on a quadrupole time-of-flight mass analyzer (6530 Agilent) to produce the fragment patterns and obtain structural information for selected biomarkers. Then, the fragmentation patterns of the biomarkers were compared to the spectral data of metabolites that had the same *m/z* in freely available databases, namely HMDB^53^, METLIN^54^, MassBank^55^ and LIPID MAPS Structure^56^. The mass tolerance between the measured *m/z* value and the exact mass of the component of interest was set to within 15 ppm. Finally, if available, confirmation with standards was carried out by comparison of retention time, isotopic distribution, and fragments of commercially available reagents (Sigma-Aldrich, St. Louis, MO) with those obtained in real samples.

## Electronic supplementary material

Supplementary Information
