# Author Correction: Large-scale Metabolomic Analysis Reveals Potential Biomarkers for Early Stage Coronary Atherosclerosis

**DOI:** 10.1038/s41598-018-23331-4

**Published:** 2018-03-19

**Authors:** Xueqin Gao, Chaofu Ke, Haixia Liu, Wei Liu, Kang Li, Bo Yu, Meng Sun

**Affiliations:** 1 Department of Cardiology, The Second Affiliated Hospital of Harbin Medical University, and The Key Laboratory of Myocardial Ischemia, Chinese Ministry of Education, Harbin, 150081 P. R. China; 2 Department of Epidemiology and Biostatistics, School of Public Health, Medical College of Soochow University, Suzhou, 215123 China; 3 Department of Epidemiology and Biostatistics, School of Public Health, Harbin Medical University, Harbin, 150081 P. R. China

Correction to: *Scientific Reports* 10.1038/s41598-017-12254-1, published online 18 September 2017

The original PDF version of this Article contained an error in the order of corresponding authors. This has now been corrected in the PDF version of this Article.

