# Supplementary material for: Large-scale Metabolomic Analysis Reveals Potential Biomarkers for Early Stage Coronary Atherosclerosis
Source: Sci Rep. 2017 Sep 18;7:11817. doi: 10.1038/s41598-017-12254-1 (PMC5603568; doi:10.1038/s41598-017-12254-1)
Supplement: Supplementary file 1 — Supplementary Information [file 41598_2017_12254_MOESM1_ESM.pdf]

Supplementary materials

Large-scale Metabolomic Analysis Reveals Potential Biomarkers for Early Stage Coronary Atherosclerosis

Xueqin Gao[a], Chaofu Ke[b], Haixia Liu[a], Wei Liu[a], Kang Li[c], Bo Yu[a], and Meng Sun[a]

[a]Department of Cardiology, The Second Affiliated Hospital of Harbin Medical University, and The Key Laboratory of Myocardial Ischemia, Chinese Ministry of Education, Harbin 150081, P. R. China.

[b]Department of Epidemiology and Biostatistics, School of Public Health, Medical College of Soochow University, Suzhou 215123, China.

[c]Department of Epidemiology and Biostatistics, School of Public Health, Harbin Medical University, Harbin 150081, P. R. China.

Address correspondence to:

Meng Sun, Ph.D., Department of Cardiology, The Second Affiliated Hospital of Harbin Medical University, and The Key Laboratory of Myocardial Ischemia, Chinese Ministry of Education, Harbin 150081, P. R. China. E-mail: sunmengzyl@163.com; and Bo Yu, M.D., Department of Cardiology, The Second Affiliated Hospital of Harbin Medical University, and The Key Laboratory of Myocardial Ischemia, Chinese Ministry of Education, Harbin 150081, P. R. China. E-mail: yubodr@163.com

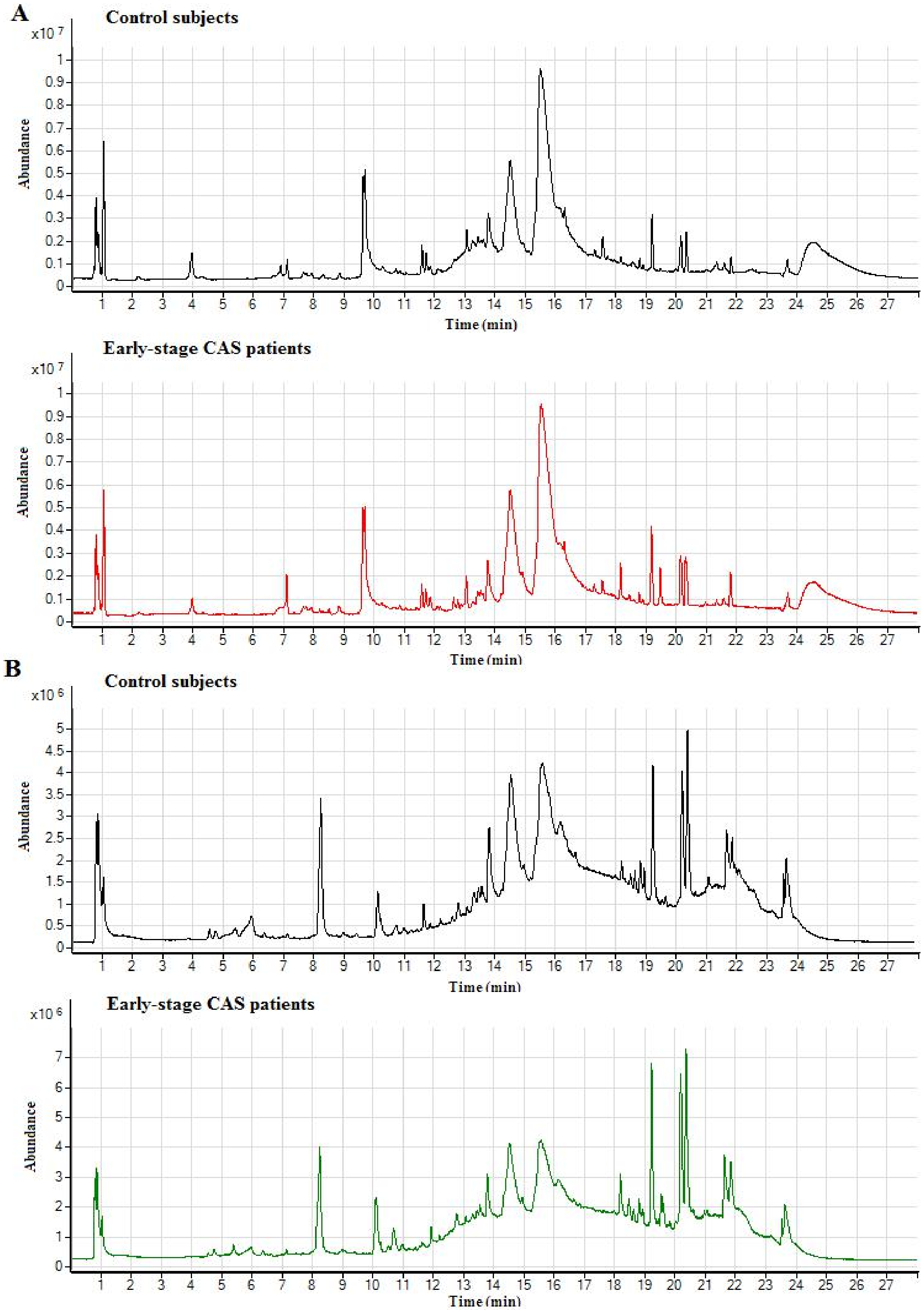

Fig. S1 Representative LC-QTOF/MS chromatograms of plasma samples for an early-stage CAS patient and a control subject acquired in (A) positive mode and (B) negative mode.

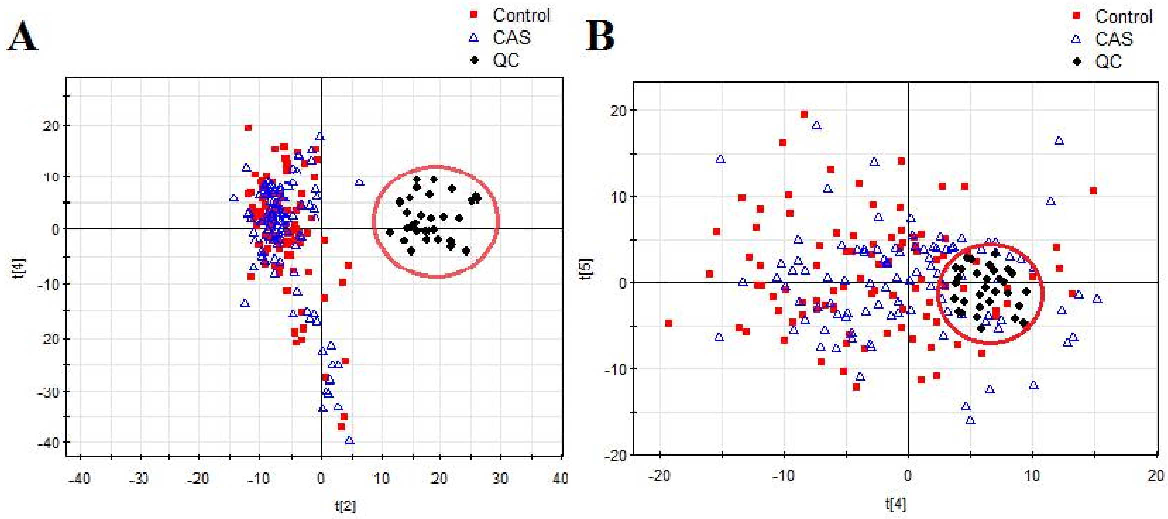

Fig. S2 PCA score plot for discriminating early-stage CAS patients and controls in (A) the ESI+ mode; (B) the ESI- mode.

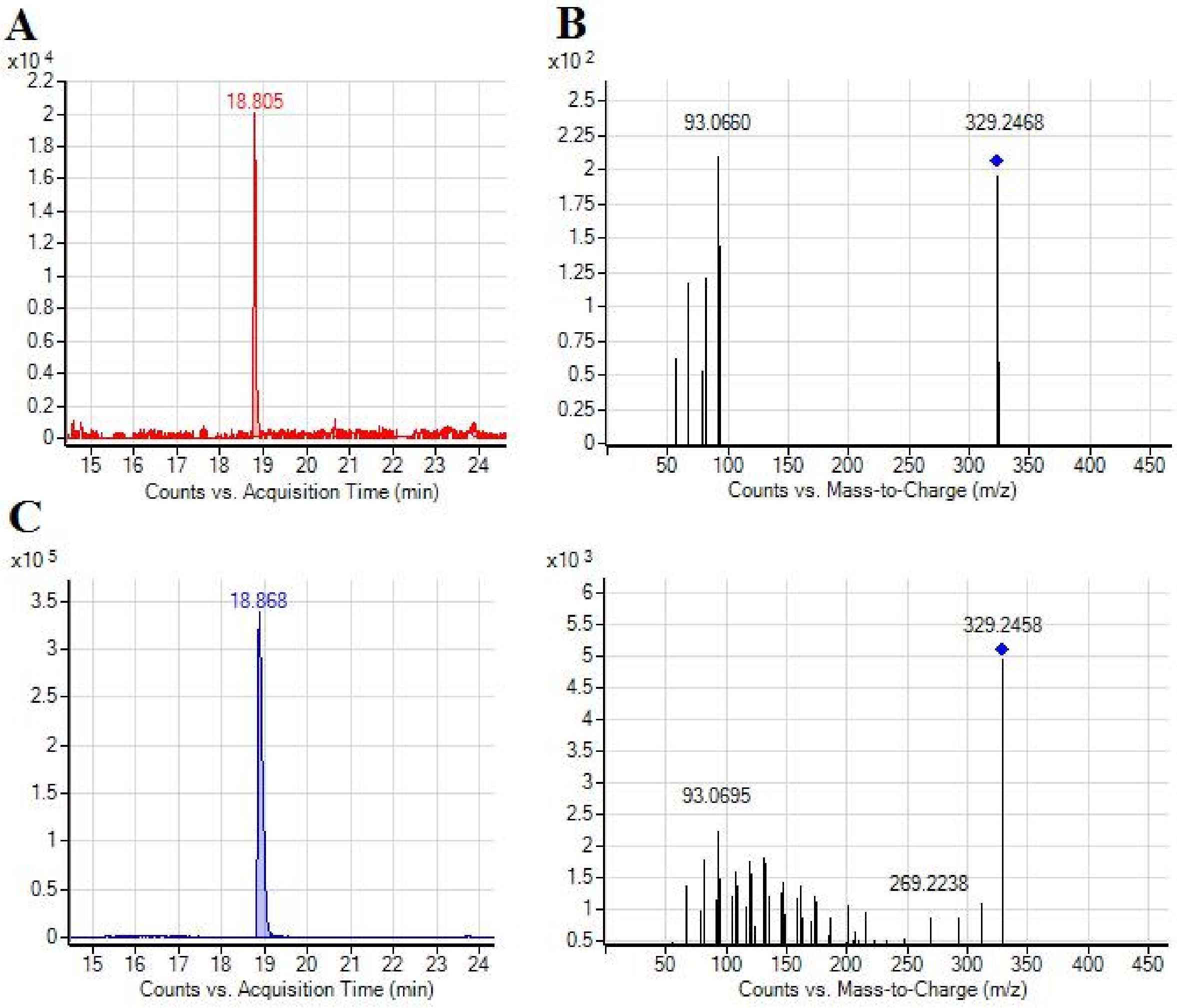

Fig. S3 The identification information of potential biomarker docosahexaenoic acid in positive mode. (A) The

extracted ion chromatographic peak of quasi-molecular ion ($[M+H]^+$) at m/z 329.2445 in a plasma sample. (B) The MS/MS spectrum of quasi-molecular ion ($[M+H]^+$) at m/z 329.2445 at 18.83min in a plasma sample. (C) The extracted ion chromatographic peak of the reference standard of docosahexaenoic acid. (D) The MS/MS spectrum of the reference standard of docosahexaenoic acid.

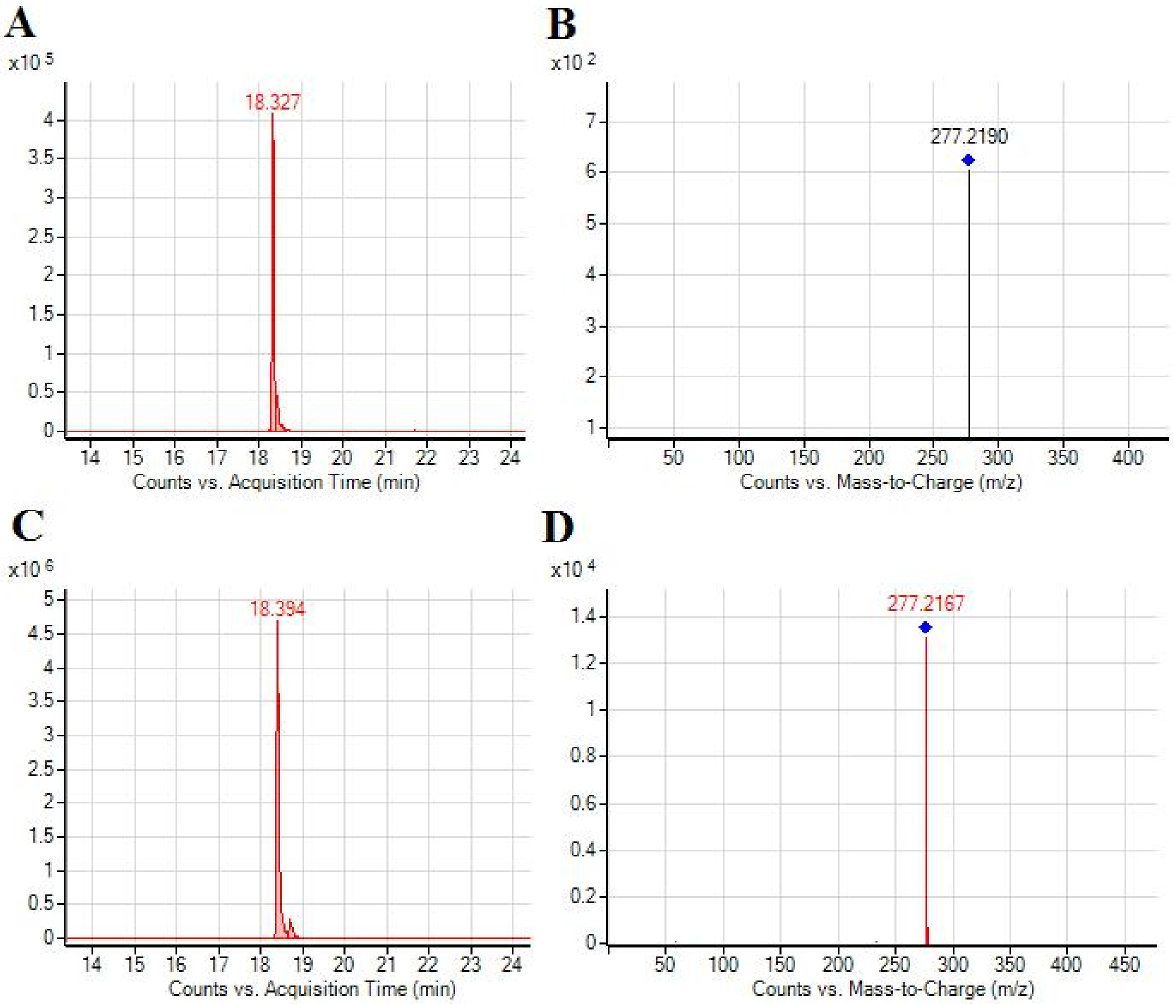

Fig. S4 The identification information of potential biomarker Linolenic Acid in negative mode. (A) The extracted ion chromatographic peak of quasi-molecular ion ($[M-H]^-$) at m/z 277.2165 in a plasma sample. (B) The MS/MS spectrum of quasi-molecular ion ($[M-H]^-$) at m/z 277.2165 at 18.30min in a plasma sample. (C) The extracted ion chromatographic peak of the reference standard of Linolenic Acid. (D) The MS/MS spectrum of the reference standard of Linolenic Acid.

A

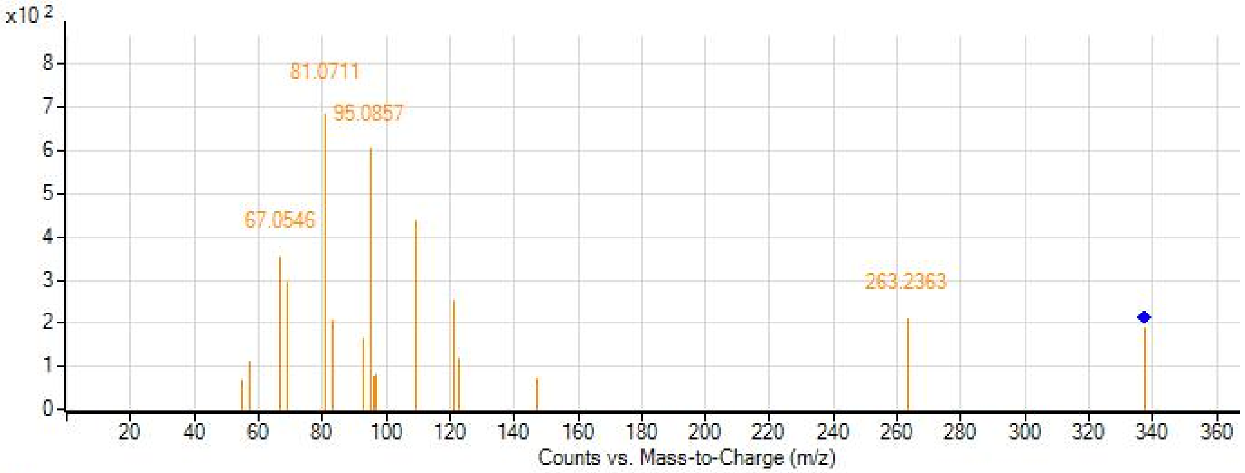

B

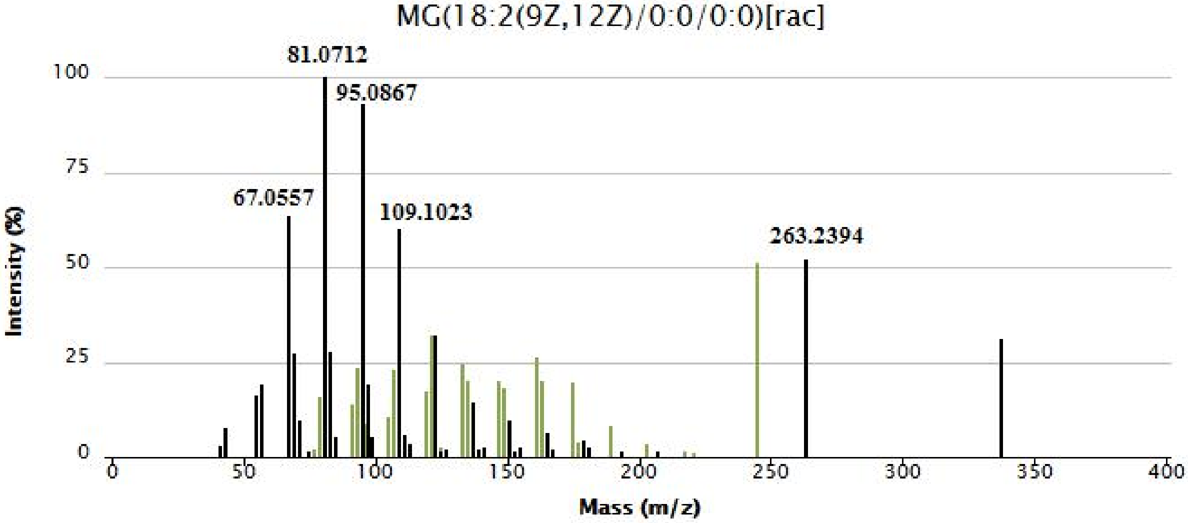

Fig. S5 The identification information of potential biomarker MG(18:2) in ESI+ mode. (A) The MS/MS spectrum of quasi-molecular ion ([M+NH4]+) at m/z 372.3085 at 18.18min in a plasma sample. (B) The MS/MS spectrum of MG(18:2) in the Metlin database.

A

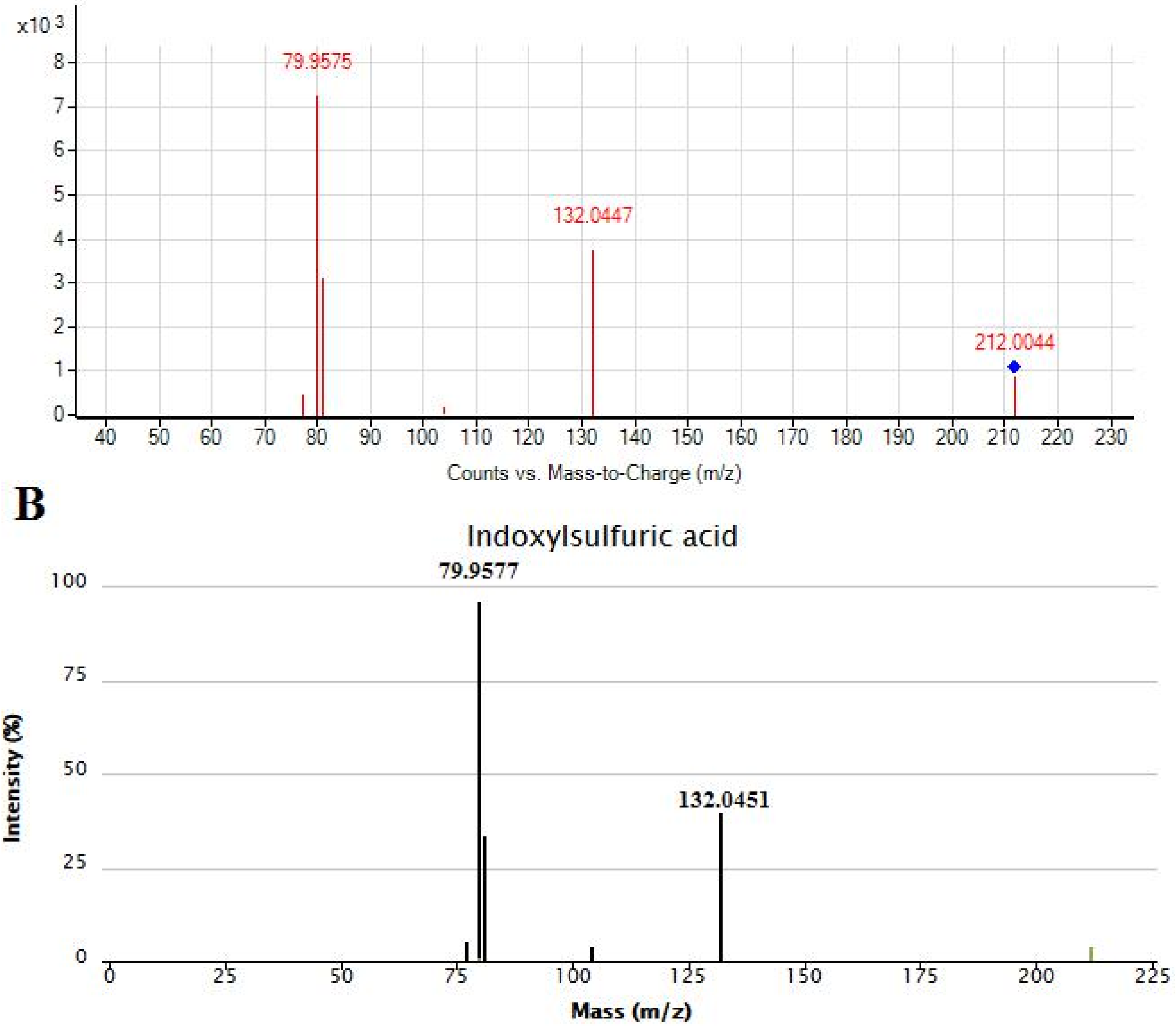

Fig. S6 The identification information of potential biomarker Indoxylsulfuric acid in ESI- mode. (A) The MS/MS spectrum of quasi-molecular ion ([M-H]-) at m/z 212.0045 at 5.52 min in a plasma sample. (B) The MS/MS spectrum of quasi-molecular ion ([M-H]-) of Indoxylsulfuric acid in the Metlin database.

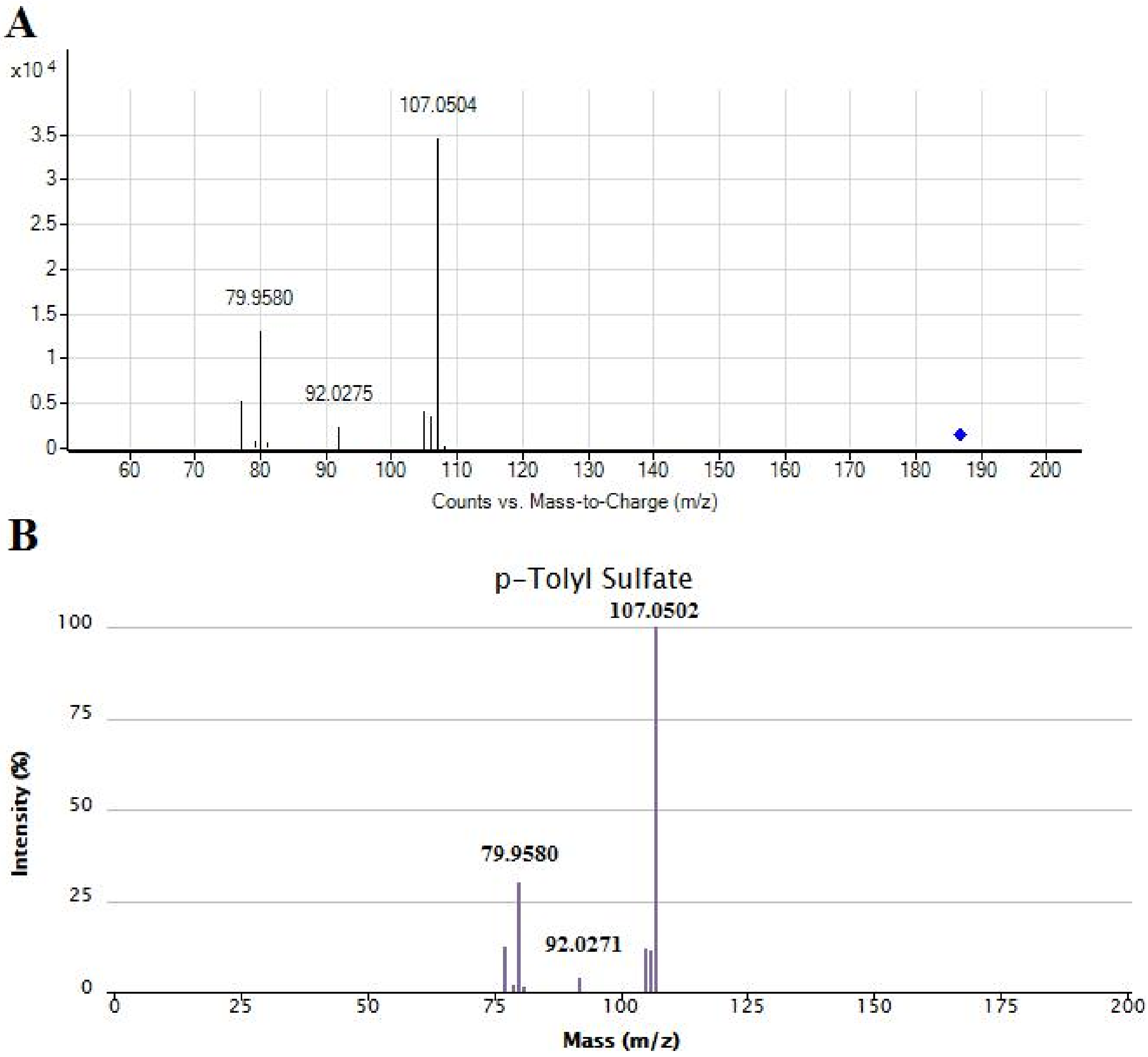

Fig. S7 The identification information of potential biomarker p-Tolyl Sulfate in ESI- mode. (A) The MS/MS spectrum of quasi-molecular ion ([M-H]-) at m/z 187.0065 at 6.45min in a plasma sample. (B) The MS/MS spectrum of quasi-molecular ion ([M-H]-) of p-Tolyl Sulfate in the Metlin database.

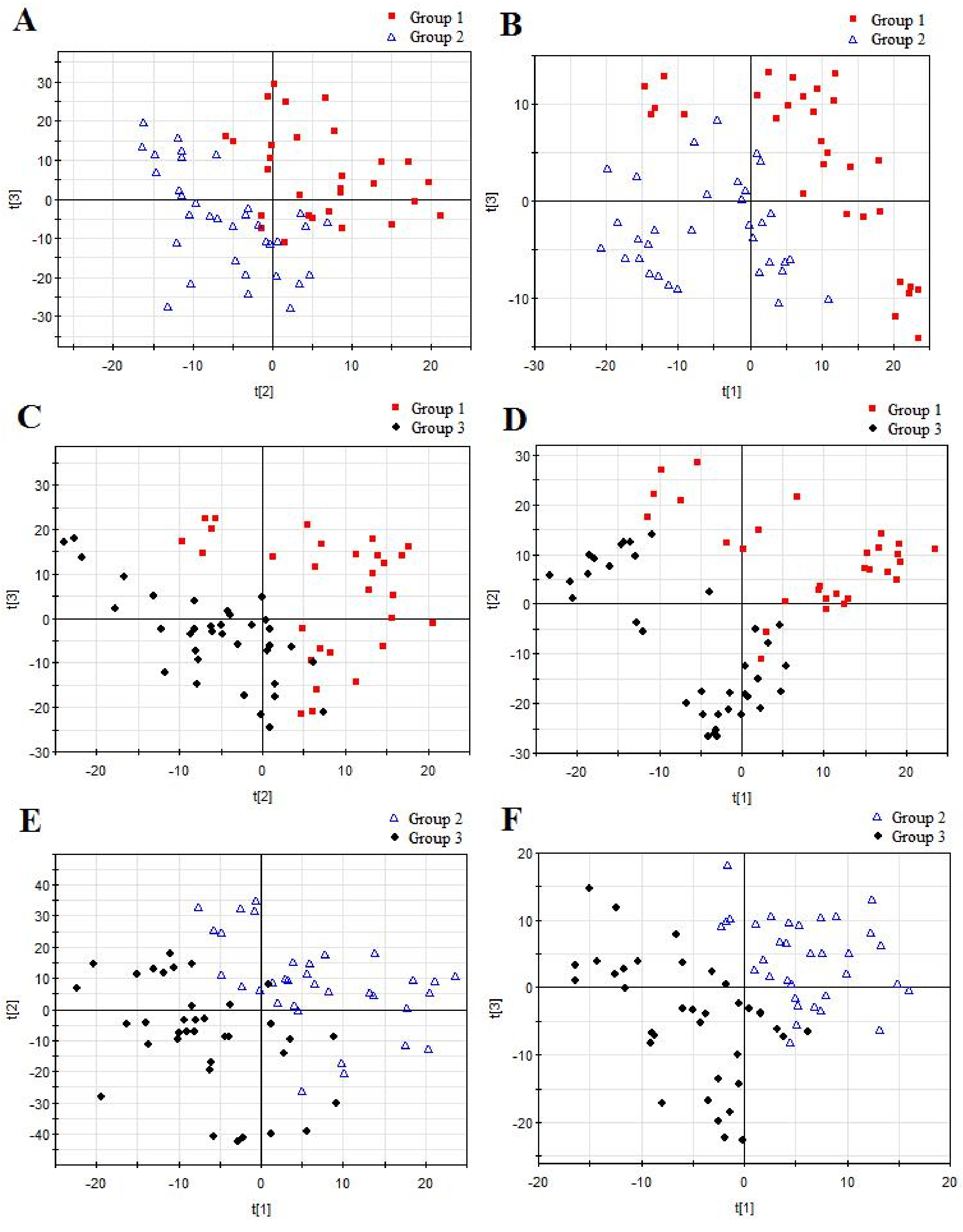

Fig. S8 PLS-DA scores plots between each two groups in combined training and test set samples. (A,B) group1 vs group2; (C,D) group1 vs group3; (E,F) group2 vs group3 in the ESI+ and ESI- mode, respectively.

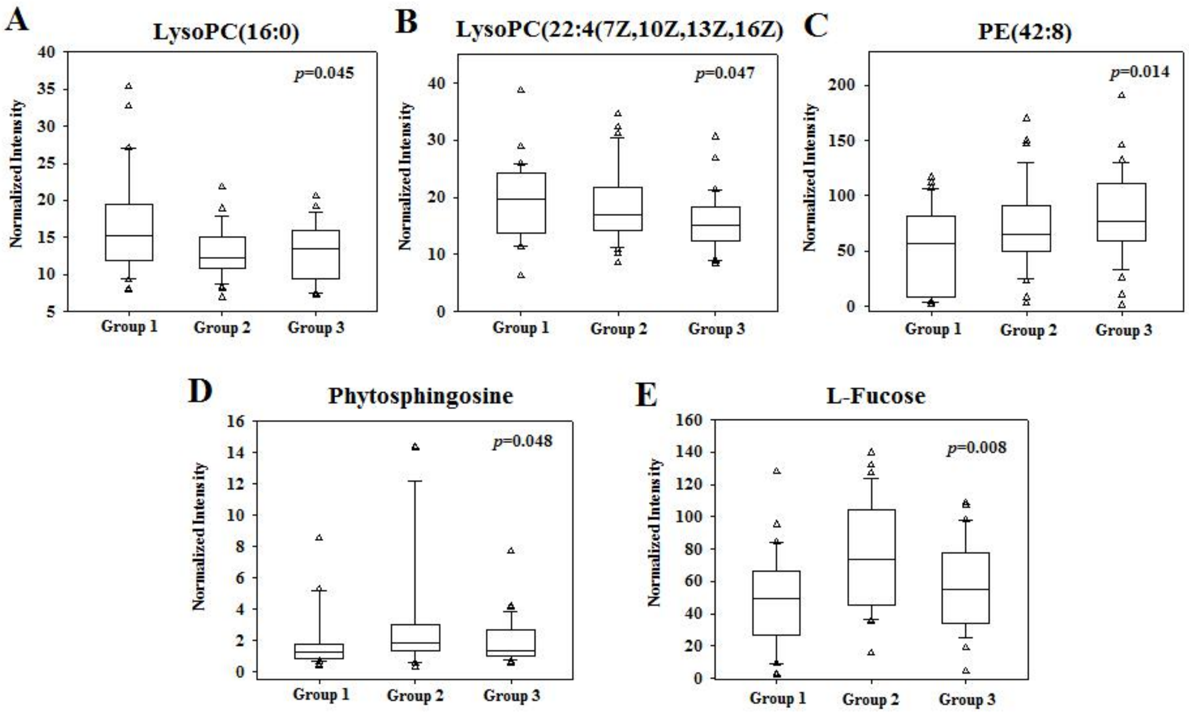

Fig. S9 Box plots of 5 metabolites that significantly changed in plasma samples of early CAS patients with different stages. The box has three lines at 25% quartile, median, and 75% quartile with extended whiskers to show the 5th and 95th percentiles of data. The symbols ‘ Δ ’ are used as the outliers.

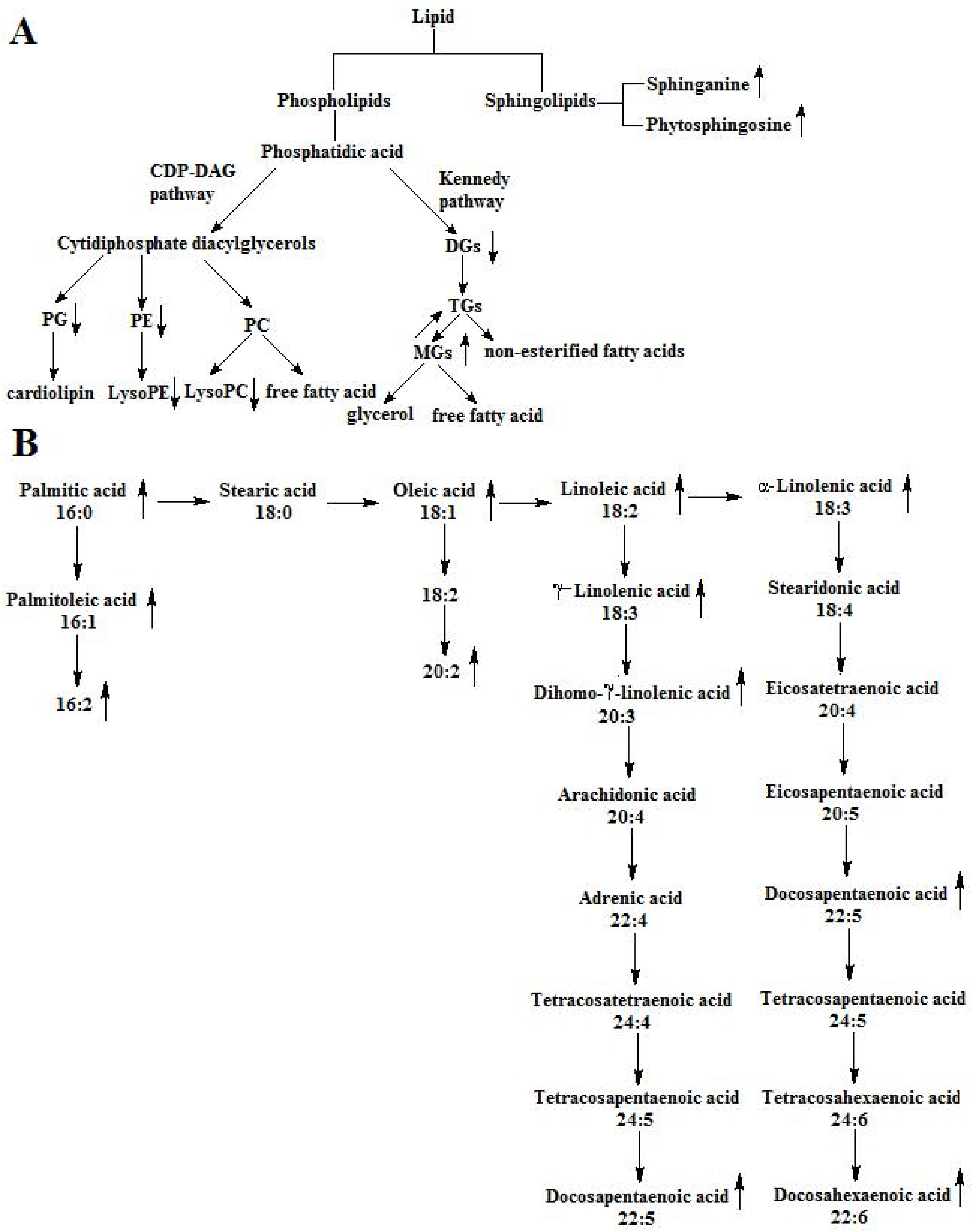

Fig. S10 Disturbed metabolic pathways associated with the formation and development of early stage CAS. (A) lipid metabolism; (B) long-chain fatty acid synthesis. Up arrows demonstrate up-regulated and down arrows mean down-regulated in the early-stage CAS patients compared to controls.

Table S1 The association between clinical characteristics and 42 metabolic signatures using Pearson correlation analysis

Marker	age	TC	TG	LDL	HDL	apoA	apoB	hs-CRP	CK	CK-MB	Troponin I	Ejection fraction
V01	0.0120	-0.0589	-0.0668	0.0336	0.1669	0.2270	0.0046	-0.2038	-0.1288	-0.0587	0.0392	-0.1134
	0.8664	0.4149	0.3548	0.6416	0.0200	0.0022	0.9510	0.0105	0.0720	0.4188	0.5936	0.1373
V02	0.0422	-0.0547	0.0073	0.0525	0.1645	0.2753	0.0836	-0.1306	-0.1808	-0.1918	0.0823	-0.0885
	0.5528	0.4491	0.9193	0.4682	0.0219	0.0002	0.2645	0.1029	0.0112	0.0077	0.2615	0.2472
V03	0.0716	-0.0461	0.0431	-0.0603	-0.0085	-0.0256	-0.0221	-0.0070	0.0788	0.0466	-0.0067	-0.0155
	0.3139	0.5233	0.5504	0.4033	0.9059	0.7329	0.7685	0.9304	0.2722	0.5212	0.9275	0.8396
V04	-0.0558	-0.0165	0.0990	0.0171	-0.1071	-0.0470	0.0385	-0.0267	0.0672	0.0321	-0.0773	0.0336
	0.4325	0.8195	0.1695	0.8127	0.1371	0.5313	0.6079	0.7396	0.3497	0.6589	0.2919	0.6604
V05	0.0328	-0.0454	-0.0519	0.0591	0.0921	0.2324	0.1494	-0.0570	-0.1371	-0.1487	-0.0941	0.0140
	0.6444	0.5297	0.4720	0.4134	0.2017	0.0017	0.0453	0.4780	0.0553	0.0396	0.1992	0.8548
V06	0.1013	-0.0886	0.0647	-0.0627	0.1289	0.1839	0.0137	-0.1619	-0.1499	-0.1698	-0.0472	-0.1262
	0.1536	0.2192	0.3702	0.3849	0.0733	0.0135	0.8552	0.0428	0.0360	0.0185	0.5203	0.0982
V07	-0.0385	-0.0942	0.1985	-0.0410	-0.0552	0.0413	-0.0690	-0.0530	0.0319	-0.0063	-0.0297	-0.0721
	0.5880	0.1916	0.0055	0.5703	0.4443	0.5816	0.3573	0.5095	0.6570	0.9304	0.6861	0.3456
V08	0.0462	-0.0584	0.0093	0.0481	0.1438	0.2130	0.0607	-0.1157	-0.1492	-0.1820	0.0979	-0.0704
	0.5163	0.4190	0.8974	0.5055	0.0454	0.0041	0.4186	0.1491	0.0368	0.0115	0.1816	0.3576
V09	-0.0136	-0.1140	0.0224	0.0214	0.0254	0.1461	-0.1200	-0.1539	-0.0208	-0.0689	-0.0332	-0.0867
	0.8484	0.1135	0.7563	0.7674	0.7248	0.0504	0.1087	0.0543	0.7724	0.3423	0.6507	0.2567
V10	0.1168	-0.0443	-0.0912	0.0974	-0.1261	-0.1582	-0.0147	-0.0893	-0.0057	0.0067	0.2519	0.0050
	0.0997	0.5394	0.2061	0.1768	0.0797	0.0339	0.8452	0.2662	0.9363	0.9265	0.0005	0.9482
V11	-0.0276	-0.0149	0.0523	0.0448	0.0500	-0.1074	0.0856	-0.0351	0.0644	-0.0153	-0.0612	0.0154
	0.6982	0.8366	0.4691	0.5350	0.4883	0.1514	0.2534	0.6629	0.3698	0.8334	0.4040	0.8403
V12	0.0459	-0.1117	-0.1112	-0.0380	0.1423	0.1139	0.0405	0.0073	-0.1136	-0.1038	0.0542	-0.0679
	0.5191	0.1212	0.1225	0.5992	0.0478	0.1281	0.5894	0.9280	0.1129	0.1518	0.4598	0.3751
V13	-0.0540	0.1391	-0.1195	-0.0464	-0.0404	-0.0322	0.0483	-0.0126	-0.0952	-0.0351	-0.0417	0.1161
	0.4478	0.0530	0.0969	0.5206	0.5762	0.6676	0.5197	0.8757	0.1846	0.6290	0.5703	0.1281
V14	-0.0536	0.1293	-0.0684	-0.0095	-0.0527	0.0575	-0.0475	-0.0443	-0.0257	0.0117	-0.0593	0.0598
	0.4511	0.0723	0.3435	0.8950	0.4656	0.4433	0.5269	0.5820	0.7210	0.8717	0.4187	0.4342
V15	0.0328	0.0065	-0.0603	0.0734	-0.2056	-0.2567	0.0220	-0.0302	0.0106	0.0807	0.0705	-0.0629
	0.6446	0.9289	0.4039	0.3088	0.0040	0.0005	0.7691	0.7074	0.8829	0.2656	0.3366	0.4107
V16	0.0230	-0.0194	-0.0340	0.0326	-0.1564	-0.1433	-0.0310	-0.0195	0.0318	0.0290	0.0708	-0.0394
	0.7468	0.7887	0.6375	0.6519	0.0294	0.0550	0.6795	0.8081	0.6582	0.6900	0.3340	0.6069

Marker	age	TC	TG	LDL	HDL	apoA	apoB	hs-CRP	CK	CK-MB	Troponin I	Ejection fraction
V17	0.1125	-0.0262	-0.0976	0.0853	-0.0864	-0.0922	-0.0252	-0.0814	-0.0344	0.0391	0.2889	0.0016
	0.1129	0.7173	0.1758	0.2370	0.2308	0.2183	0.7368	0.3111	0.6321	0.5904	<0.0001	0.9833
V18	0.1008	-0.0333	-0.1214	-0.0928	0.03045	0.0754	0.0281	0.1534	-0.1605	-0.0707	0.0664	0.0410
	0.1554	0.6451	0.0917	0.1983	0.6734	0.3141	0.7077	0.0552	0.0246	0.3296	0.3652	0.5923
V19	-0.0652	0.1535	-0.0831	-0.1060	0.0350	0.0867	0.0478	-0.0361	-0.0088	-0.0741	0.0433	-0.0166
	0.3589	0.0327	0.2495	0.1412	0.6284	0.2469	0.5240	0.6532	0.9024	0.3069	0.5554	0.8286
V20	0.0440	-0.0365	0.0353	0.0034	-0.0259	-0.0533	-0.0503	0.1402	0.1014	0.0832	0.1712	0.1365
	0.5362	0.6134	0.6251	0.9628	0.7201	0.4771	0.5025	0.0799	0.1574	0.2512	0.0188	0.0733
V21	-0.0088	-0.0301	-0.0345	0.0163	0.0038	0.0319	0.0181	0.0177	-0.1015	-0.0949	-0.1209	-0.0989
	0.9019	0.6769	0.6326	0.8211	0.9580	0.6705	0.8094	0.8257	0.1570	0.1903	0.0983	0.1956
V22	-0.0371	-0.0048	-0.0253	0.0072	-0.0495	0.0225	0.0708	-0.0691	-0.0821	-0.0929	-0.1342	-0.0948
	0.6021	0.9471	0.7258	0.9206	0.4931	0.7640	0.3451	0.3895	0.2529	0.1998	0.0664	0.2148
V23	-0.0226	-0.0331	0.0757	-0.1016	-0.1196	0.0001	0.0873	-0.1049	0.1050	0.0938	-0.0789	0.0232
	0.7509	0.6468	0.2939	0.1588	0.0969	0.9999	0.2439	0.1911	0.1429	0.1958	0.2820	0.7616
V24	0.0005	-0.0699	0.0119	0.0061	0.0215	-0.1156	0.0033	-0.0458	-0.0540	-0.0421	0.0325	0.0590
	0.9944	0.3331	0.8688	0.9333	0.7661	0.1224	0.9649	0.5689	0.4519	0.5619	0.6577	0.4405
V25	-0.0565	-0.0986	0.0223	-0.0195	-0.0682	-0.1571	-0.0160	-0.0073	-0.0100	0.0281	0.1092	0.1144
	0.4268	0.1716	0.7576	0.7877	0.3445	0.0352	0.8312	0.9279	0.8890	0.6992	0.1357	0.1338
V26	0.0360	-0.0276	0.0209	0.0639	-0.0696	-0.1383	0.0660	-0.0142	0.0383	0.0467	0.0815	0.1002
	0.6127	0.7029	0.7723	0.3763	0.3351	0.0642	0.3790	0.8599	0.5943	0.5202	0.2664	0.1899
V27	0.0203	-0.0028	-0.0295	0.0799	-0.0437	-0.0849	0.0565	0.0150	-0.0245	-0.0445	0.0702	0.0666
	0.7752	0.9689	0.6832	0.2683	0.5454	0.2573	0.4510	0.8523	0.7335	0.5395	0.3382	0.3841
V28	-0.0238	-0.0903	-0.0355	-0.0194	-0.0551	-0.0919	0.0309	-0.0575	0.0117	0.0468	0.0971	0.1097
	0.7377	0.2104	0.6229	0.7887	0.4455	0.2199	0.6801	0.4741	0.8704	0.5189	0.1848	0.1510
V29	-0.0035	-0.0396	0.0452	0.0470	-0.0126	-0.1060	0.0676	-0.0162	0.0034	0.0520	0.0833	0.0854
	0.9611	0.5839	0.5315	0.5150	0.8619	0.1567	0.3672	0.8400	0.9622	0.4739	0.2557	0.2638
V30	-0.1200	-0.0882	-0.0120	0.0419	-0.0254	-0.0805	0.0015	-0.0129	0.0324	-0.0094	0.0261	0.0764
	0.0906	0.2214	0.8684	0.5615	0.7255	0.2826	0.9840	0.8724	0.6518	0.8973	0.7227	0.3178
V31	-0.0920	-0.0109	0.0167	0.0362	-0.0013	-0.0531	-0.0061	0.0326	-0.0222	0.0151	0.0281	0.0696
	0.1952	0.8805	0.8174	0.6164	0.9861	0.4794	0.9352	0.6854	0.7579	0.8349	0.7017	0.3631
V32	-0.0393	-0.0663	-0.0199	0.0200	-0.0620	-0.0907	0.0131	-0.0449	0.0214	0.0899	0.1097	0.0946
	0.5805	0.3581	0.7834	0.7821	0.3902	0.2258	0.8614	0.5770	0.7664	0.2151	0.1342	0.2159
V33	-0.0236	-0.0741	0.0637	-0.0209	-0.0780	-0.1697	0.0147	-0.0393	0.0131	0.0722	0.0936	0.0998
	0.7403	0.3047	0.3773	0.7724	0.2798	0.0228	0.8446	0.6255	0.8550	0.3196	0.2014	0.1912

Marker	age	TC	TG	LDL	HDL	apoA	apoB	hs-CRP	CK	CK-MB	Troponin I	Ejection fraction
V34	0.0470	0.0553	0.0478	0.1377	0.0753	0.0152	0.3075	-0.0347	-0.0721	-0.0666	-0.0098	-0.0038
	0.5085	0.4441	0.5084	0.0555	0.2968	0.8393	<0.0001	0.6663	0.3154	0.3590	0.8934	0.9605
V35	-0.0691	-0.0719	-0.0012	0.0527	-0.0478	0.1535	-0.1078	-0.0465	0.0276	-0.1011	0.0115	-0.1037
	0.3307	0.3193	0.9867	0.4657	0.5082	0.0397	0.1496	0.5634	0.7006	0.1630	0.8758	0.1747
V36	-0.0721	-0.0852	0.0595	-0.0690	-0.0174	0.0397	0.0256	-0.2073	-0.0305	-0.0162	0.0575	-0.0855
	0.3101	0.2377	0.4097	0.3393	0.8095	0.5963	0.7335	0.0092	0.6714	0.8231	0.4335	0.2635
V37	-0.0043	-0.0251	0.0724	-0.0975	-0.0625	-0.0763	-0.0155	0.0160	0.0832	0.1059	0.0997	0.1009
	0.9524	0.7279	0.3159	0.1763	0.3866	0.3085	0.8364	0.8427	0.2463	0.1439	0.1734	0.1865
V38	-0.0816	0.1868	0.0136	0.0864	0.0994	-0.1130	0.0728	0.0003	-0.1007	-0.0110	0.0152	0.0414
	0.2508	0.0091	0.8503	0.2311	0.1681	0.1308	0.3317	0.9973	0.1603	0.8796	0.8364	0.5890
V39	-0.0121	0.1817	-0.0388	0.1203	0.0099	-0.0837	-0.0478	0.0399	0.0519	-0.0781	-0.0472	0.0331
	0.8648	0.0112	0.5916	0.0948	0.8909	0.2638	0.5242	0.6199	0.4701	0.2815	0.5205	0.6651
V40	0.0287	0.0510	0.0220	0.1232	0.0613	0.0082	0.2247	-0.0551	-0.0786	-0.0600	0.0044	-0.0175
	0.6869	0.4803	0.7604	0.0870	0.3958	0.9133	0.0024	0.4930	0.2735	0.4082	0.9523	0.8194
V41	0.1340	-0.1048	0.0008	-0.0622	-0.0652	-0.1411	-0.0430	-0.0287	-0.0580	-0.0020	0.0397	0.0994
	0.0586	0.1459	0.9917	0.3891	0.3662	0.0588	0.5666	0.7216	0.4193	0.9781	0.5890	0.1933
V42	0.0819	0.0567	0.0401	0.1507	0.0516	-0.0095	0.2865	-0.0343	-0.0710	-0.0643	-0.0117	-0.0083
	0.2488	0.4324	0.5790	0.0360	0.4752	0.8989	<0.0001	0.6698	0.3225	0.3758	0.8738	0.9138

Top was Pearson correlation coefficient, and bottom was *p*-value calculated from Pearson correlation analysis. Markers relate to the metabolites labelled in Table 2 for both positive and negative modes

Table S2 Plasma differential unidentified ions for discriminating early-stage CAS patients from controls

Marker	RT(min)	*m/z*	*VIP*[a]	*p*-Value[b]	FC[c]	AUC	RSD (%)[d]
Positive electrospray ionization mode (ESI+)							
1	5.46	195.0896	1.1684	0.0205	0.57	0.6243	19.31
2	12.97	298.2724	1.0334	0.0414	-0.31	0.6094	7.05
3	5.22	117.1019	1.5606	0.0226	-0.4	0.6222	15.55
4	21.85	779.5335	1.7399	0.0276	0.24	0.6181	13.11
Negative electrospray ionization mode (ESI-)							
5	6.45	189.0042	1.3489	0.0188	0.8	0.6204	15.46
6	11.77	448.3049	1.2976	0.0038	-0.62	0.6484	26.50
7	18.15	417.3000	1.1663	0.0469	0.26	0.6018	18.86
8	19.23	463.3407	1.6785	0.0108	0.28	0.6306	16.92
9	13.09	365.2357	1.2866	0.0395	0.91	0.6053	26.82
10	13.19	216.1698	1.6530	0.0129	0.97	0.6273	23.80
11	16.01	583.3115	1.4334	0.0360	-0.17	0.6074	26.54
12	20.14	299.2575	1.9409	0.0174	0.16	0.6218	15.77
13	18.83	438.2648	1.4562	0.0312	0.48	0.6104	21.19
14	11.72	449.3282	1.3047	0.0030	-0.6	0.6519	21.27

[a] Variable importance in the projection (VIP) was obtained from PLS-DA with a threshold of 1.0. [b] The p-value was calculated from the nonparametric Kruskal-Wallis rank sum test. [c] Fold change was calculated as a binary logarithm of the arithmetic mean ratio between patients vs controls, where a positive value indicates that a relatively higher concentration present in patients while a negative value means a relatively lower concentration as compared to the control subject. [d] Variation of the biomarker concentrations in the quality control samples expressed as relative standard deviation (RSD%)

Abbreviations: Retention time (RT, min);Measured mass to charge ratio (*m/z*); Variable importance in the projection (VIP); Fold change (FC); The area under the ROC curve (AUC)

Table S3 The association between clinical characteristics and differential unidentified ions using Pearson correlation analysis

RT (min)	*m/z*	TC	TG	LDL	HDL	apoA	apoB	hs-CRP	CK	CK-MB	Troponin I	Ejection fraction
5.46	195.0896	0.0876	-0.0361	0.1152	-0.0292	-0.0255	-0.1029	0.4008	-0.0126	-0.0524	-0.0121	-0.1417
		0.2248	0.6178	0.1098	0.6865	0.7336	0.1692	<0.0001	0.8613	0.4704	0.8688	0.0630
12.97	298.2724	-0.0868	0.0456	-0.0358	0.2670	-0.0150	0.1861	0.0581	-0.0712	-0.0084	-0.0587	-0.1588
		0.2288	0.5276	0.6205	0.0002	0.8414	0.0124	0.4696	0.3212	0.9081	0.4239	0.0369
5.22	117.1019	-0.1155	-0.0587	-0.0823	-0.0239	0.0436	-0.0087	0.0524	0.0540	0.0302	-0.1077	-0.0435
		0.1088	0.4160	0.2542	0.7404	0.5610	0.9075	0.5148	0.4521	0.6778	0.1412	0.5696
21.85	779.5335	-0.0646	-0.02231	-0.0418	-0.0569	-0.0270	-0.1083	0.0112	-0.0277	0.1037	-0.0076	-0.0645
		0.3709	0.7575	0.5630	0.4309	0.7186	0.1477	0.8889	0.7003	0.1522	0.9175	0.3989
6.45	189.0042	0.0058	-0.0396	0.0950	-0.1046	-0.0819	-0.0920	0.0120	-0.0423	0.0393	-0.0167	-0.0625
		0.9363	0.5837	0.1877	0.1468	0.2743	0.2194	0.8817	0.5561	0.5888	0.8202	0.4143
11.77	448.3049	-0.1092	-0.0022	-0.0564	0.0169	-0.0276	-0.1184	-0.0735	0.1619	-0.0200	-0.0104	-0.0387
		0.1297	0.9761	0.4350	0.8149	0.7130	0.1135	0.3603	0.0234	0.7831	0.8876	0.6129
18.15	417.3000	-0.0285	0.1012	-0.0782	-0.0772	0.0853	-0.0637	-0.1380	0.0295	-0.0829	-0.0707	0.0227
		0.6936	0.1605	0.2784	0.2846	0.2547	0.3954	0.0848	0.6812	0.2531	0.3352	0.7666
19.23	463.3407	-0.0705	0.0641	-0.0839	-0.0536	0.1732	-0.0552	-0.1394	0.0748	-0.0272	-0.0534	-0.0200
		0.3285	0.3743	0.2446	0.4579	0.0201	0.4622	0.0817	0.2973	0.7082	0.4664	0.7941
13.09	365.2357	-0.0078	-0.0659	0.0110	-0.0687	0.0175	0.0559	-0.0293	0.1172	-0.0314	-0.0367	0.0578
		0.9146	0.3614	0.8792	0.3415	0.8157	0.4561	0.7153	0.1019	0.6654	0.6169	0.4501
13.19	216.1698	-0.0265	-0.0440	-0.0267	0.0185	0.0358	-0.0250	-0.0191	-0.0736	-0.0050	-0.0565	-0.0942
		0.7138	0.5423	0.7117	0.7976	0.6337	0.7386	0.8122	0.3053	0.9453	0.4411	0.2176
16.01	583.3115	-0.0012	0.1724	-0.1158	-0.0850	0.1592	-0.0001	-0.1536	0.2052	0.0481	-0.0782	0.0508
		0.9866	0.0162	0.1077	0.2386	0.0328	0.9988	0.0547	0.0039	0.5079	0.2862	0.5068
20.14	299.2575	-0.0736	0.0017	-0.1525	-0.0032	0.1527	0.1296	-0.0991	-0.0314	-0.0073	-0.0623	-0.0255
		0.3080	0.9807	0.0338	0.9646	0.0407	0.0828	0.2168	0.6621	0.9201	0.3957	0.7388
18.83	438.2648	-0.1059	0.0235	-0.0778	0.0186	0.2080	0.0096	-0.1015	0.1404	-0.0523	-0.0339	-0.0384
		0.1418	0.7450	0.2809	0.7966	0.0051	0.8986	0.2059	0.0497	0.4717	0.6441	0.6155

RT (min)	*m/z*	TC	TG	LDL	HDL	apoA	apoB	hs-CRP	CK	CK-MB	Troponin I	Ejection fraction
11.72	449.3282	-0.1218	-0.0061	-0.0798	0.0095	-0.0313	-0.1108	-0.0777	0.1394	-0.0169	-0.0155	-0.0669
		0.0906	0.9328	0.2689	0.8960	0.6767	0.1386	0.3336	0.0514	0.8158	0.8333	0.3816

Top was Pearson correlation coefficient, and bottom was *p*-value calculated from Pearson correlation analysis
